# A Review of Deep Learning Techniques for EEG-Based Emotion Recognition: Models, Methods, and Datasets

**DOI:** 10.12688/f1000research.171170.1

**Published:** 2025-11-18

**Authors:** P. Sreehari, U. Raghavendra, Anjan Gudigar

**Affiliations:** 1 Department of Instrumentation and Control Engineering, Manipal Institute of Technology, Manipal Academy of Higher Education, Manipal, Karnataka, 576104, India

**Keywords:** Emotion recognition, Electroencephalography, Deep neural networks, Hybrid approaches

## Abstract

Emotion Recognition (ER) with Electroencephalography (EEG) has become a major area of focus in affective computing due to its direct measurement of the activity of the brain. ER based on EEG has also advanced with the popularity of Deep Learning (DL) and its advancements related to classification accuracy and model efficiency. This systematic review is conducted following the PRISMA (Preferred Reporting Items for Systematic Reviews and Meta-Analyses) guidelines and aims to provide an overview of DL-based EEG emotion recognition approaches. A comprehensive literature search was conducted across five major databases covering the publications from 2020 to 2025. The studies with EEG signals for ER using DL architectures were included in the present review. Finally, a total of 233 articles were considered after eligibility screening. To enhance the diversity of investigation, we assessed the public datasets utilized for ER based on EEG in terms of their stimulation procedures and emotional representation. Further, the provided analysis attempts to direct future research toward EEG-based emotion identification systems that are more interpretable, generalizable, and data-efficient. This systematic review aims to provide a roadmap for developing EEG-driven ER, guiding researchers toward more reliable, scalable, and practically useful systems.

## 1. Introduction

Emotions play a vital role in Human-Computer Interaction (HCI), influencing thinking, decision-making, and social interaction. ^1^ Emotions are combinations of knowledge, behaviour, and physiological elements and have been studied for a long time to understand how people react to events that have a significant meaning for them. ^2^ The need for systems that can understand human emotion, sentiment, and cognition in various modalities has grown with the increasing popularity of affective computing. ^3^ It involves the identification and processing of human emotions and has relevant applications in health care, particularly in Computer-Aided Diagnosis (CAD) tools. ^4^ This will provide the ability to measure emotional disabilities, evaluate mental health, monitor emotional stress, and neurological disorders, such as anxiety. Affective computing will utilize bio-signals, including but not limited to EEG, facial expressions, and speech, to assess a patient’s emotional state, cognitive ability, and social functioning, facilitating diagnosis and personalized interventions. ^5, 6^ In an educational environment, emotion recognition systems facilitate the early identification of students’ self-regulation and mental health problems by analyzing their emotional expressions, allowing for timely support and intervention. ^7^

**Figure 1.**
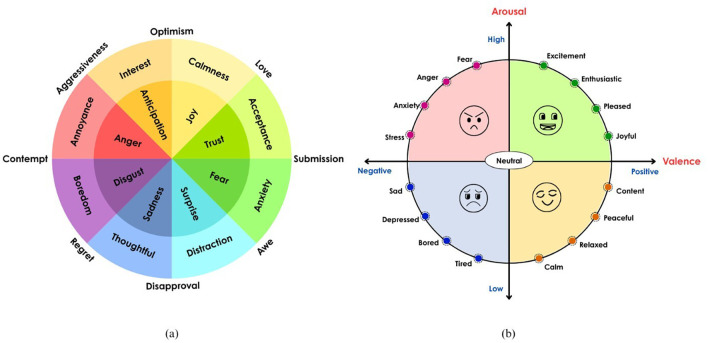
(a) Emotion wheel ^10^ (b) 2-Dimensional emotion model. ^11^

Emotion classification has evolved from categorical to dimensional theories over the years, which determines how emotional states are labeled. Ekman’s six emotions were proposed based on the cross-cultural studies of facial expressions: Fear, Surprise, Happiness, Anger, Disgust, and Sadness. ^8^ This work had a significant impact on affective computing and psychological research by demonstrating that emotions are signals developed over time rather than merely learned social norms. Plutchik proposed eight primary bipolar emotions (trust–disgust, joy–sadness, surprise–anticipation, fear–anger) arranged in a wheel-like structure, including the intensity of the emotions. ^9^ According to Plutchik, these basic states combine to form complex emotions, such as anticipation and joy, which produce optimism, as shown in Figure 1(a). ^10^ Many affective computing systems that model mixed and intensified emotional states are based on his framework. Russell proposed the valence-arousal model for emotion by positioning the emotions in a 2D space. ^11^ According to dimensional theories, emotions do not have clear labels but rather exist along a continuous psychological dimension. Valence signifies the pleasantness of the emotion, and arousal is the intensity of the emotion. This model is called the circumplex model of emotion, and Figure 1(b) illustrates basic emotions placed based on how pleasant and how intense it is. Mehrabian and Russell proposed a three-dimensional emotion model by adding the dominance dimension to the pleasure (valence) and arousal dimensions, proposing a Pleasure-Arousal-Dominance (PAD) model. ^12^ Dominance captures whether the person is in control or controlled by their emotions. Emotions such as anger and fear align strongly with these dimensions. For example, fear has a high arousal but a low dominance score, whereas anger has a high arousal and dominance score.

Depression is a mood disorder that lasts at least two weeks and causes sadness or a loss of interest or enjoyment in things that used to be fun or interesting. It makes it very hard to function and can affect sleep, appetite, focus, self-esteem, and suicidal thoughts. Over 700,000 suicides occur annually due to depression, making it a primary cause of death for individuals aged 15–29. ^13^ The World Health Organization states that nearly 4-6% of adults (about 332 million individuals worldwide) will face depression at some point and a higher prevalence in women (around 6%) compared to men (around 4%). ^14^ The COVID-19 pandemic worsened problems with anxiety, sadness, stress, and loneliness. WHO indicates that one in seven adults suffers from a mental health disorder (accounting for 10% of the overall disease burden). A study in India revealed that 65-75% of adolescents pursuing higher education in Tier-I cities are facing moderate to severe depression or anxiety amid the pandemic. Furthermore, the pandemic has greatly escalated India’s already growing youth suicide rate. Among youngsters, suicide is the fourth leading cause of death (accounting for 1 in 100 deaths globally). ^15– 17^

### 1.1 Automated emotion recognition system

**Figure 2.**
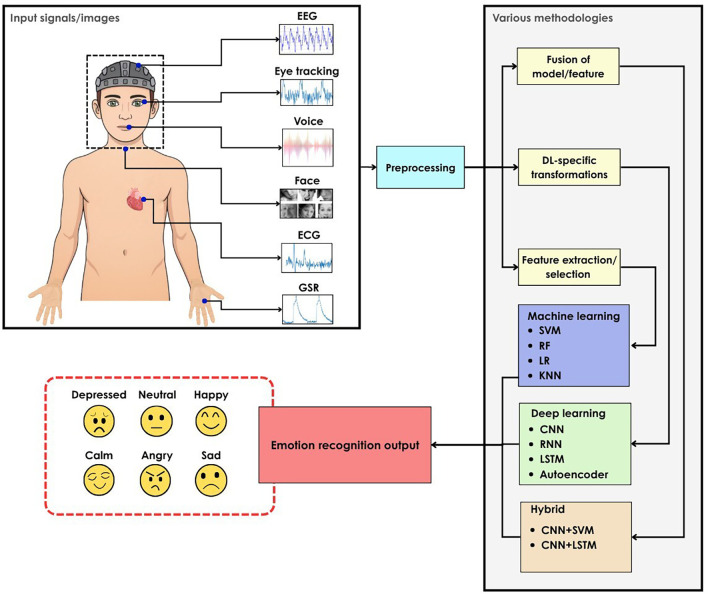
Methods of emotion recognition.

Automated Emotion Recognition (AER) systems, typically implemented as CAD systems, are important because emotional states can significantly influence human actions and decisions. Automated ER has endless applications in HCI, healthcare, applied learning, driving assistance, marketing, and education. ^18^ AER can perform an objective and consistent analysis that was difficult through manual observation alone. ^19^ Various Machine Learning (ML) and Deep Learning (DL) methods are employed to process the data from multiple sources to understand the emotion. Hybrid methods are also used for better performance, ^19^ as illustrated in Figure 2.

Researchers have conducted numerous studies to distinguish emotions using different modalities to aid the diagnosis, monitoring, and treatment plans. The modalities include non-physiological signals such as speech signal, ^20, 21^ facial expression, ^22, 23^ and text data ^24, 25^ along with physiological signals, such as Galvanic Skin Response (GSR), ^26, 27^ Electrocardiogram (ECG), ^28, 29^ EEG, ^30, 31^ and eye movement signals, ^32, 33^ based emotion recognition also gained popularity as it directly indicates an individual’s emotional state. Since physiological signals have a direct impact from the emotional stimulation, a method for emotion recognition using the recorded physiological signals has a superiority over non-physiological data. It is effective for people who cannot speak or express their feelings externally. The individual cannot control the signal produced in the body by emotional stimulation. ^34^ EEG is an effective way to capture the emotional state of a person because it provides a real-time insight into the voltage fluctuations in the brain caused by stimulation. ^35^ Various frequency bands in the brainwave signal ( *α*, *β*, *γ*, *θ*, and *δ*) correspond to different emotional states like anger, fear, happiness, sadness, and surprise. ^35^ Since EEG signals directly reflect brain dynamics, they are considered more reliable than facial expression or speech for emotional recognition. ^36^ Numerous studies follow a multimodal approach to emotion classification by combining two or more physiological or non-physiological modalities. Deep learning facilitates smooth information fusion across modalities in such systems. EEG combined with any other modality, such as face, ^37^ eye movement, ^33^ speech, ^38^ and any other physiological signal, ^39, 40^ has been shown to obtain better classification results.

**1.1.1 Various deep learning models used for emotion recognition**

Traditional machine learning techniques such as k-Nearest Neighbors (KNN), ^41^ Random Forest (RF), ^42^ Logistic Regression (LR), ^43^ Support Vector Machine (SVM), ^44^ and Decision Trees (DT) ^45^ were initially used for emotion classification, which require careful manual feature extraction and feature selection. ^46^ Since it demands significant domain expertise, it could produce biased and subjective results. ^46^ Recently, deep learning techniques - especially different variants of Convolution Neural Networks (CNNs), ^47^ Recurrent Neural Networks (RNNs), ^48^ and hybrid models ^49^ have become popular since they are capable of learning complex patterns and extracting the most relevant features from the raw data, resulting in significant improvements in the performance of the AER system. Each modality has its own advantages and limitations, but physiological signals generally provide a more reliable way to determine emotional states. However, these signals are challenging to process and are highly prone to external noise. On the other hand, speech- and facial expression–based methods are less complex, but since they can be intentionally hidden or manipulated by the subject, accurate analysis becomes difficult. ^18^ Multimodal approaches combining data from different sources (e.g., EEG-face, audio-video) are often recommended to enhance the performance. ^50^

EEG-based ER has a wide set of advantages by offering objective and real-time insight into emotional states, which is a positive aspect in terms of healthcare, mental health, and human-computer interaction. ^51^ EEG registers brain activity directly, making it trustworthy in contexts that rely on authenticity. Because of the complexity of analyzing the signal manually, machine learning models fail to perform very well on the manually extracted features. Recent developments in the EEG domain have focused on using deep learning techniques and multi-modal integration to increase accuracy and practicality. Attention mechanism and Transfer Learning (TL) techniques have significantly helped in the advancement of EEG-based emotion recognition by improving the feature extraction and model generalization processes. The attention mechanism allows the model to emphasize the most informative EEG channels, frequency bands, and brain regions. ^52– 54^ This extraction of key features is essential for distinguishing emotions. TL techniques allow a model trained on one dataset to adapt to new subjects or new datasets, addressing the individual differences in EEG signals. ^55^

### 1.2 Motivation and contributions of this paper

Recently, several research studies have been performed in the field of emotion recognition. Liu et al. ^56^ conducted a study on EEG-based Multimodal Emotion Recognition (EMER), integrating EEG with other biosignals such as Electromyogram (EMG) and ECG for emotion classification. The review by Gkintoni et al. ^57^ discusses DL techniques, including CNNs and RNNs, with a focus on feature extraction methods and their relevance to real-world applications. Erat et al. ^58^ presents deep learning methods as part of classification strategies within the Brain-Computer Interface (BCI) pipeline for EEG-based ER systems. Wang et al. ^34^ offers a comprehensive review of deep learning models such as CNNs, Deep Belief Networks (DBN), and RNNs, emphasizing their roles in emotion recognition. In a study, the authors categorize DL approaches into CNNs, RNNs, and hybrid models, and also discuss different evaluation strategies such as subject-dependent and subject-independent testing. ^59^ Several other studies highlight the importance of multimodal integration, the utilization of deep learning for automatic feature extraction, and the design of complete EEG emotion recognition pipelines. ^60, 61^ Geng et al. ^62^ reviewed the DL methods based on the feature learning method used in the studies into single, attention-based, domain adaptation-based, and hybrid DL models.

**Figure 3.**
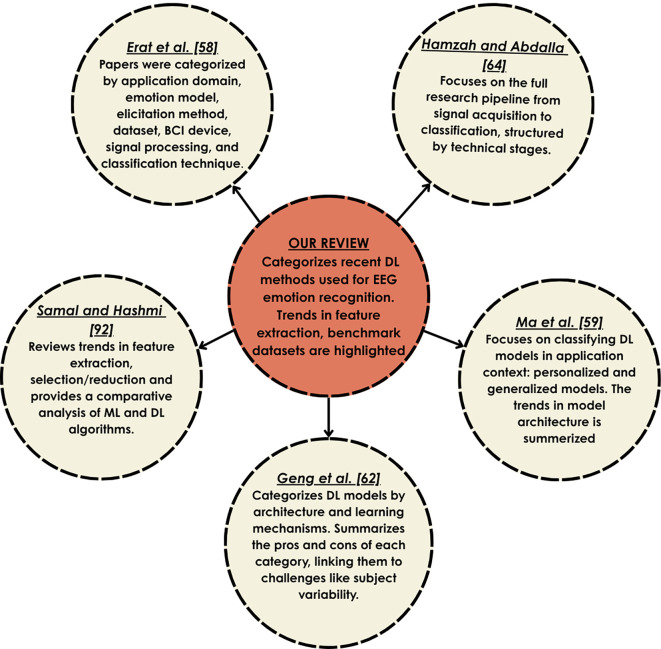
Comparison of our review with existing studies.

There are a number of clear limitations, even though the reviewed papers offer helpful information about EEG-based emotion recognition. Several papers offer broad overviews without clearly categorizing models based on DL paradigms (supervised, unsupervised, or hybrid), which limits methodological clarity. Some reviews treat DL as part of a larger AI discussion, leading to a lack of focus on DL-specific end-to-end pipelines or evaluation strategies. Only a few studies provide a structured classification of DL approaches and discuss subject-independent evaluations. Moreover, eXplainable AI (XAI), ^63^ or any other type of model interpretability methods, needs to be addressed, which are critical for building transparent and trustworthy emotion recognition systems. Furthermore, real-time applicability, computational cost, and ethical concerns should also be given importance since they are more important at the practical implementation stage. Figure 3 shows a comparison of our study with the existing reviews on EEG-based ER.

Studies on emotion recognition based on EEG are essential because they offer a direct understanding of the electrical activity of the brain during emotion elicitation, providing a unique, objective, and temporally accurate insight into brain dynamics that no other mode of emotion recognition can offer. ^51^ Although multimodal approaches are used to improve accuracy, focusing on EEG alone helps to understand the neural mechanisms of emotion, develop interpretable models, and address the challenges specific to brain signal processing. ^64^ Noise and inter-subject variability are two primary challenges encountered in studies based on EEG signals. EEG signals are less prone to intentional masking or manipulation of emotion compared to facial and voice modalities. Moreover, EEG-based studies have driven innovations in DL, feature extraction, and real-time tracking of emotions, contributing to the foundation of multimodal emotion recognition. Thus, the main objectives of the proposed study are as follows:

• Perform a systematic review of the recent studies on EEG-based emotion recognition, highlighting the methodologies, datasets, and evaluation strategies.
• Explore various DL architectures employed in EEG-based emotion recognition, focusing on their strengths, limitations, and performance patterns.
• Provide the roadmap for developing an efficient and robust emotion recognition system.

This paper performs a systematic literature review following the PRISMA (Preferred Reporting Items for Systematic Reviews and Meta-Analyses) guidelines, ensuring transparency. ^65, 66^ The review includes peer-reviewed articles selected from five large scientific databases and screened according to the inclusion and exclusion criteria. The main advantages of the proposed study are as follows:

• The study identifies and analyzes how the recent advancements in DL techniques have been applied in the emotion recognition domain.
• DL strategies are categorized into supervised, unsupervised, and hybrid approaches to examine how different DL paradigms handle complex EEG signals and how they represent emotions.
• Additionally, the EEG emotion datasets used by various studies were also analyzed in terms of stimuli, emotion annotation levels, and input modalities.
• The review also discusses challenges faced in this field, such as noise handling, individual variability in the captured signals, and generalization issues.
• In this study, future research directions are also proposed, focusing on improving the generalization of the model and the diversity of the databases.

The paper is organized into the following sections: Section 2 outlines the search strategy followed for identifying and selecting relevant studies. Section 3 presents an analysis of various EEG datasets and the preprocessing techniques commonly used in emotion recognition research. Section 4 discusses DL architectures employed for EEG-based ER systems, highlighting their methodologies, performance, and limitations. Section 5 presents the key challenges and future directions in this domain. Finally, Section 6 concludes the paper with a summary of key insights, followed by standard sections on author contributions, funding information, conflict of interest, data availability, and references.

## 2. Search strategy

A systematic literature search process was used to locate studies relevant to this review across five major databases: PubMed, Scopus, Embase, ProQuest, and Web of Science. The objective of the searches was to gather recent and relevant literature in the field of emotion recognition based on EEG that uses deep learning frameworks. Rayyan AI, a web-based tool for systematic reviews, was used to organize, filter, and screen the search results after they were filtered. ^67^

The search included articles published from January 2020 to March 2025. These articles were generally focused on DL models and advances in EEG data processing, particularly related to emotion recognition. All databases were accessed through their respective institutional portals.

### 2.1 Search terms and queries

**Figure 4.**
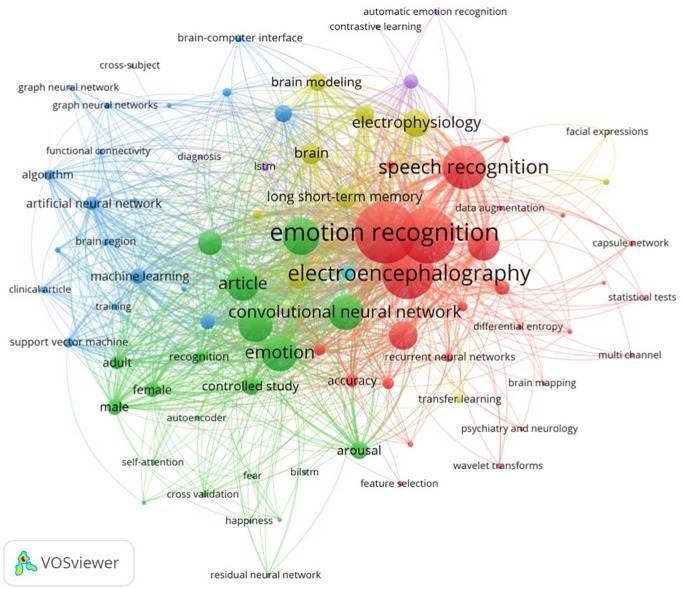
Keyword co-occurrence network visualized using VOSviewer. ^68^

A search strategy was formulated using specific keywords. The co-occurrence of keywords in the selected studies is represented as network clusters using the VOSviewer tool in Figure 4. ^68^ The terms were grouped into three core categories, including:

• **EEG-related terms:** “EEG”, “electroencephalogram”, “electroencephalography”
• **Emotion-related terms:** “emotion recognition”, “affective computing”, “emotion identification”, “emotion classification”
• **Deep learning methods:** “deep learning”, “neural network”

Boolean operators were used (e.g., (“EEG” OR “electroencephalography”) AND (”emotion recognition” OR “emotion identification”) AND (“deep learning” OR “neural network”)) to formulate the queries. The results were restricted to journal articles published between 2020 and 2025, omitting review papers in databases where this option was available.

### 2.2 Selection of papers for the review

**Figure 5.**
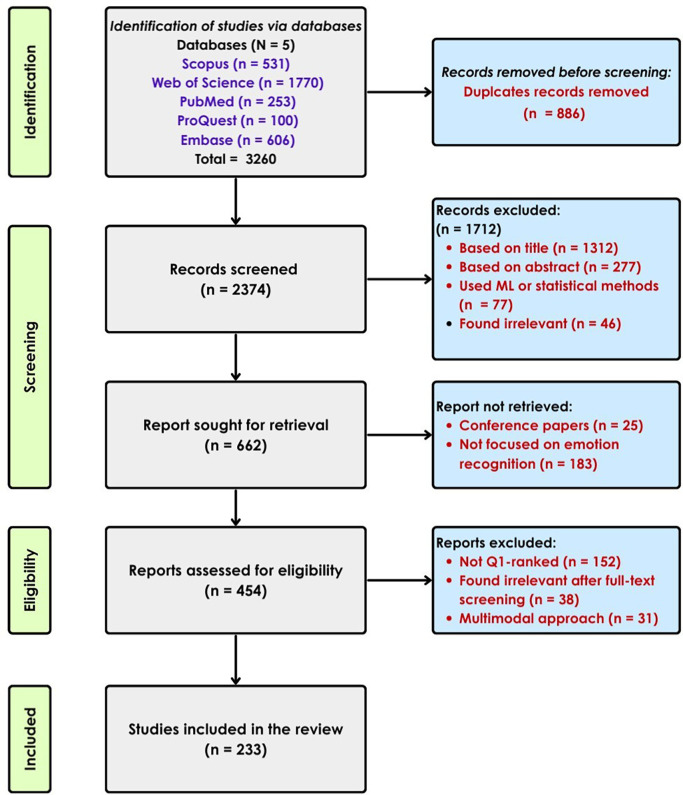
Selection of articles using PRISMA guidelines.

The initial search yielded a total of 3260 records across all databases. The records were imported to Rayyan AI for the ease of screening and management. Rayyan’s automated deduplication tools identified and removed 886 records, resulting in 2374 unique records. Title and abstract screening were carried out by one reviewer based on the inclusion criteria. Full-text screening was performed on the articles that passed the screening and excluded the articles that did not meet the criteria. A PRISMA 2020 flow diagram, which demonstrates the article selection process is shown in Figure 5.

The following criteria were applied to select studies for inclusion in this review:

• The study must use DL techniques to classify or recognize emotions.
• The focus should be primarily on EEG signals, either alone or combined with other modalities (e.g., facial expressions, physiological data).
• The research must involve original experimental studies, and the EEG signal should be a central data source for emotion recognition.
• Only articles published in Q1-ranked journals (as per Scopus) were considered to ensure high-quality peer-reviewed content.

The following studies were excluded from the review:

• Emotion recognition using on ML approaches without the use of DL.
• Research that used EEG benchmark datasets for purposes other than emotion recognition (e.g., motor imagery or cognitive workload).
• Review or survey papers.
• Studies published in journals not classified as Q1 based on the most recent Scopus or journal ranking data.

Due to the broad nature of the search query, many irrelevant records were retrieved from the databases, many of which were not EEG-based studies. Rayyan AI’s automated resolution tool removed 886 duplicates, and 2374 unique articles were initially screened. Title and abstract were used for the initial screening process. Many articles were found ineligible, 1712 to be exact, because they did not align with the primary objective of the study. There were 219 articles marked as “maybe” in the Rayyan screening tool; however, 25 out of these were conference papers and were removed. 183 papers were excluded because the papers were later found to be not using EEG as the primary modality. After this, 454 articles were initially shortlisted and 152 articles were removed for not being Q1-ranked after additional eligibility criteria were applied. After full-text screening for significance, 38 more articles were removed from consideration, and 31 papers were excluded for using a multimodal approach. This resulted in a final list of 233 articles to be included in this review.

## 3. Analysis of various datasets and preprocessing techniques

**Figure 6.**
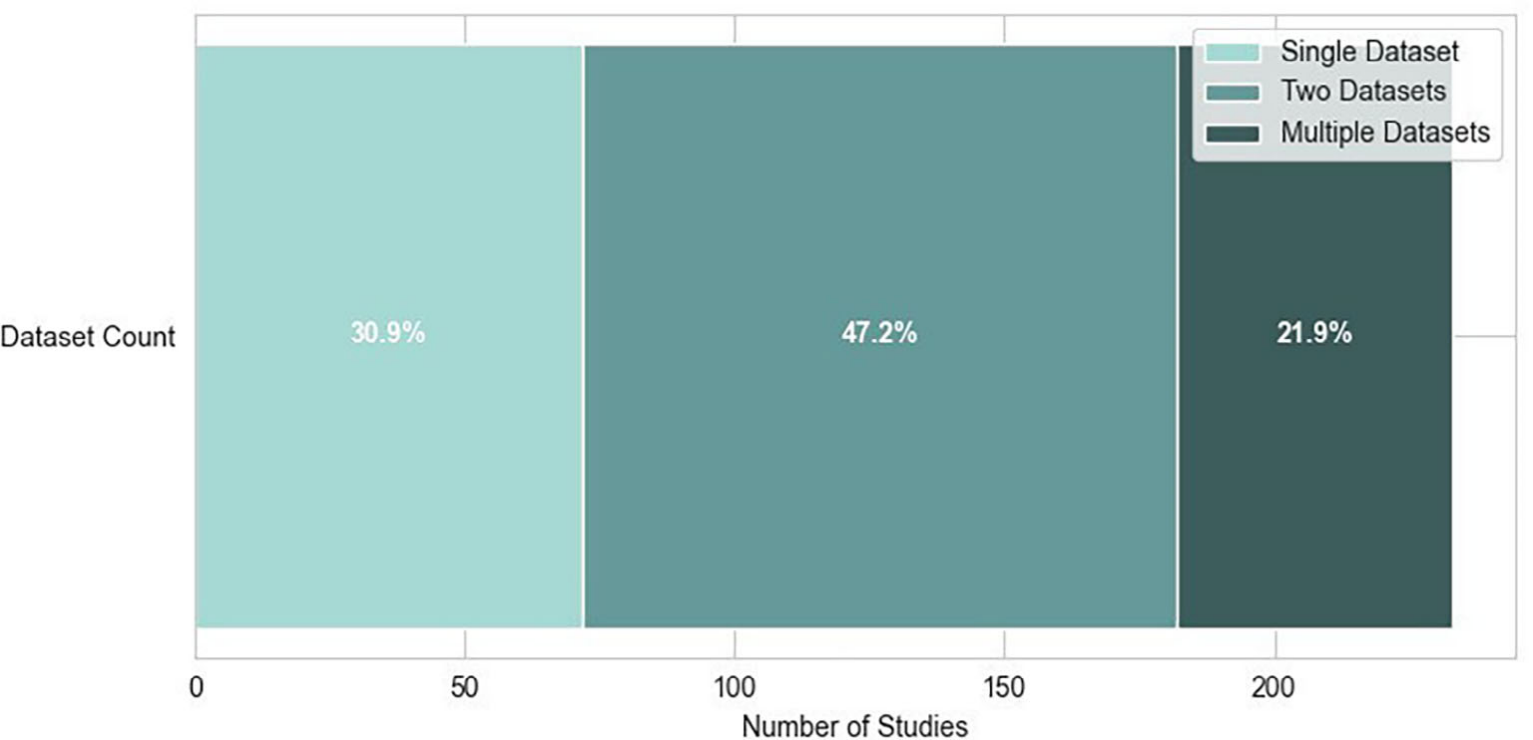
Distribution of dataset usage in studies.

Researches in emotion recognition are supported by the growing availability of well-annotated datasets. These datasets use video or film clips, audio, and images as emotional stimuli and provide EEG recordings collected under controlled experimental settings. They capture brain activity while the subject experiences different emotions, allowing researchers to train and evaluate ML or DL models for emotional state classification. Table 1 (refer to extended data) shows common datasets that are used in EEG-based ER studies. ^69– 88^ Most of the datasets also include recordings from multiple modalities, along with EEG and self-assessment reports from the subjects. This section presents an overview of the most commonly used EEG emotion datasets, describing their design, emotional stimuli, and emotion labelling mechanisms used. Most of the studies use more than one dataset for ensuring the robustness of the model among different subjects, devices, stimuli, and environments. Figure 6 shows the usage of different numbers of datasets among the selected articles.

In EEG emotion datasets such as DEAP, participants self-report their emotions immediately after each stimulus using the Self-Assessment Manikin (SAM), a pictorial 1–9 scale that measures arousal, valence, and dominance. These self-assessment ratings provide emotional ground truth labels, which can be mapped to discrete classes, or they can also be used directly as inputs in regression models. ^69^

**Figure 7.**
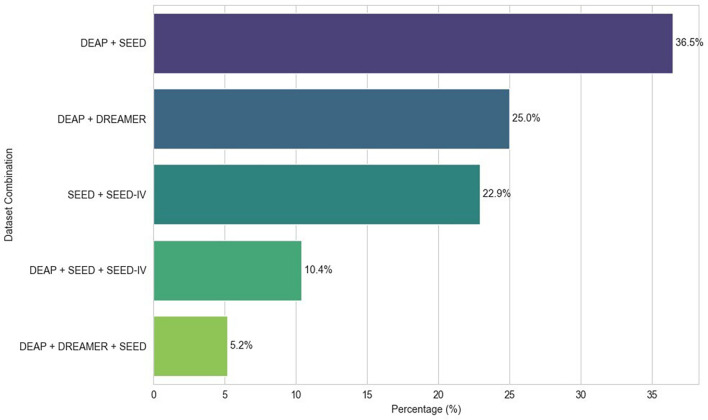
Most common dataset combinations.

EEG signals are highly prone to noise, and for obtaining meaningful brain activity, preprocessing is essential. Recorded EEG signals are often affected by ECG, EMG, eye blink, and power-line interferences. To facilitate an accurate emotion recognition performance, several preprocessing steps are performed to increase the quality of the EEG signals. Common preprocessing techniques include bandpass filtering (typically 0.5-45 Hz) to filter out the physiological artifacts, ^89^ and some studies employ artifact removal techniques such as Independent Component Analysis (ICA) or manually address the issues like eyeblinks and muscle movements. ^90, 91^ The downsampling operation is often performed on the captured signals to remove the artifacts. The DEAP data set sampled at a frequency of 512 Hz is then down-sampled to 128 Hz, and a 4-45 Hz bandpass filter is applied to remove EOG artifacts. ^92^ In the SEED dataset, EOG was also recorded, which was later used to identify the eyeblink artifacts from the EEG data. ^71^ A bandpass filter between 0.3 and 50 Hz was used for artifact removal and the downsampling to 200 Hz, which is later segmented into 1-second non-overlapping epochs. Frequency decomposition is also performed using wavelet or Fourier transform to isolate the signals into standard EEG bands, such as alpha(α), beta(β), gamma(γ), delta(δ), and theta(θ). ^93^ Differential Entropy (DE) features are calculated across the standard frequency bands, and sessions are padded to a fixed length to ensure consistency. ^70^ The authors used Artefact Subspace Reconstruction (ASR) on the DREAMER dataset to deal with high-variance noise that remained after band-pass filtering, such as blinks or muscle activity. ^72^ ASR employs a sliding-window Principal Component Analysis (PCA), in which the system dynamically identifies and interpolates any signal components whose variance exceeds a threshold. In the AMIGOS dataset, ^78^ EEG samples were sampled to 128 Hz, average-referenced, and high-pass filtered at 2 Hz, removing eyeblink artifacts using blind-source-separation method. ^94^ Power Spectral Density (PSD) features were calculated using the Welch method with 1-second windows (128 samples) between 3-47 Hz, later averaged into five frequency bands. In EEG-based ER research, the use of datasets varies depending on the type of learning paradigm applied. Figure 7 shows the different dataset combinations (more than 2 datasets) in the selected articles.

In a study, EEG signals from hearing-impaired subjects were collected in positive, negative, and neutral emotional states using movie clips as stimulation. A downsampling operation (200 Hz) was performed and band-pass filter (1-75 Hz) was used along with a trap filter (49-51 Hz) to remove the industrial frequency signal. ICA was used to remove the artifacts of oscillogram and myoelectricity to improve the quality. ^95^

## 4. DL architectures for EEG-based ER

In human interactions, emotions play a major role, influencing decision-making and behaviour. ^96^ The need for systems that can understand human emotion, sentiment, and cognition across various modalities has grown with the increasing popularity of affective computing. ^34^ Various modalities such as facial expressions, speech, EEG, GSR, ECG, heart rate, and eye movements are used to understand emotional states; among them, physiological signals like EEG, ECG, and GSR are particularly valuable as they directly reflect a person’s emotional state. ^57^

### 4.1 Affective computing with EEG

EEG-enabled emotion detection has gained popularity in recent years, as EEG provides insight into the brain activities induced by certain stimuli, measured using scalp electrodes. ^35^ It captures the voltage fluctuations in the brain caused by the neuron interactions, providing real-time insight into the emotional state. Various emotional states, such as happiness, sadness, anger, fear, and surprise, correspond to different frequency bands in brainwave signals, including *α*, *β*, *γ*, *θ*, and *δ.* Since EEG signals directly reflect brain dynamics, they are considered more reliable than facial expression or speech for emotional recognition. ^36^ Unfortunately, EEG signals are often noisy and high-dimensional with individual differences across people, which makes the manual analysis challenging and limits the traditional feature engineering methods. ^97, 98^ Thus, an advanced computational method is needed to extract useful information from raw EEG signals, using which an accurate emotion classification can be done.

Deep Neural Networks (DNNs) are advanced computational models inspired by the structure and functional similarities of the human brain. In contrast to traditional algorithms, DNNs are trained on large datasets, enabling them to make accurate predictions by learning more complex patterns in the data. ^99^ A DNN consists of layers of interconnected nodes known as neurons: an input layer to capture data, multiple hidden layers to process and transform data, and an output layer to generate final output. The ability of neurons to receive, process, and forward information facilitates the learning of complex patterns in the data. ^100^ The input layer captures unprocessed data such as images, text, and audio. The input is transformed by several neurons in the hidden layers using computations that identify various patterns. Parameters such as weights and biases are optimized during backpropagation by iteratively updating the network to reduce prediction errors. The final output, derived from these internal computations, is produced through the node in the output layer.

**Figure 8.**
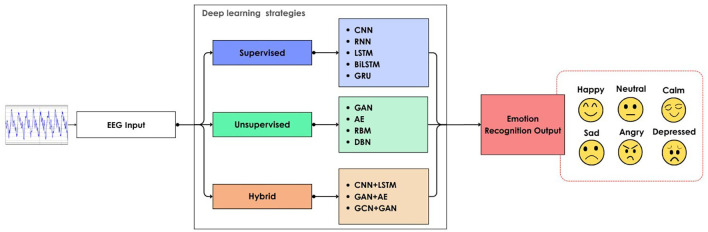
DNN models for EEG emotion recognition.

Different DL strategies are followed for classifying emotion using EEG signal, as shown in Figure 8. Supervised methods work well with datasets with reliable labelling. The unsupervised method is suitable for identifying hidden patterns in an unlabeled dataset. The hybrid method combines unsupervised and supervised methods, or two supervised methods, for better results. This flexibility of DNN makes it suitable for emotion recognition using the EEG signal.

EEG emotion recognition has evolved over the years with DL. Zheng et al. ^101^ presented a Deep Belief Network (DBN) trained on the DE features of multichannel EEG to classify positive versus negative emotional states. The DBN achieved accuracies of 86.91%, and a combination of the DBN with the Hidden Markov Model (HMM) provided an accuracy of 87.62%, outperforming the traditional ML methods such as SVM and KNN, which require manual feature engineering. Jirayucharoensak et al. ^31^ introduced a DNN structure that integrates PCA-based covariate shift correction for improved performance in emotion analysis using EEG signal. Using a stacked autoencoder deep network with the PSD as input, PCA chooses the best components. Liu et al. ^102^ explored the application of CNNs by converting EEG data into image-like representations after feature extraction. Subsequent research on hybrid deep learning models for EEG emotion recognition frequently references their work, which showed the potential of CNNs in this area.

### 4.2 Supervised DNNs

In supervised DNNs, the model is trained using input signals paired with their corresponding emotion labels (e.g., happy, sad, etc.), and during training, its internal parameters are iteratively adjusted to minimize the difference between actual and predicted emotional states. This is usually done by changing the network’s parameters via the backpropagation algorithm. Supervised DNNs can be further categorized based on the specific task they are designed to perform and the type of data they process.

Multi-layer Perceptron (MLP) is a basic feedforward DNN model that uses non-linear activation functions and backpropagation. ^103^ In EEG emotion recognition, MLPs are primarily used to analyse the emotional state using the handcrafted DE and PSD features. Li et al. ^104^ used a Hierarchical 3D MLP-based Neural Network (HMNN) for cross-subject emotion recognition. HMNN uses 3D-MLP Blocks for multi-period EEG feature extraction and fusion. The study highlights individual differences in brain activity that produce high variance in accuracy between subjects. Unlike CNN and RNN, MLPs are not capable of capturing spatial and temporal dependencies effectively, yet they serve as a simple and computationally efficient baseline model for EEG emotion classification.

Building on the idea of learning from limited or unlabeled data, self-supervised EEG emotion recognition explores the unsupervised nature of EEG signals by creating pretext tasks that models need to solve using pseudo labels derived from the data itself. This facilitates the extraction of robust and transferable representations. For example, models such as EEGFuseNet use a hybrid CNN-RNN-GAN network to capture deep spatio-temporal features for EEG, learning with both reconstruction and adversarial features without labels. ^105^ The clustering of emotion states is done afterwards using unsupervised clustering (e.g., hypergraph partitioning). Other works, such as Generative Adversarial Network-based Self-supervised data augmentation (GANSER) , employ self-supervision in the form of adversarial training and masking-based tasks, pre-training the network over a large amount of unlabeled data before the supervised classification. ^106^ These methods significantly reduce the need for manual annotations and improve the model’s capabilities of generalization with respect to unseen subjects.

Semi-supervised approaches for EEG-based emotion recognition leverage limited labeled data and large amounts of unlabeled examples to achieve efficient learning and potential transferability. Methods such as Semi-supervised and Domain Adversarial learning with EEG (SEDA-EEG) ^107^ are based on two-stage training: first, a supervised method is followed to train the model using labeled source domain data, and then it is fine-tuned using domain adversarial learning and pseudo-labeling for the unlabeled target domain data in the absence of the true labels. The pseudo labels obtained from the feature representations of the target are then used in adaptation by combining supervised and unsupervised signals. Another example is Semi-Supervised Domain Adaptation (SSDA) framework, which uses just a few labeled samples per class of each new subject combined with many unlabeled recordings to align feature and prediction distributions. ^108^

**4.2.1 Convolutional Neural Networks (CNN)**

LeCun et al. ^47^ first proposed a backpropagating neural network that learned from 16×16 raw grayscale images without any manual feature engineering. This helped in the development of trainable convolution filters and weight-sharing, which later laid the foundation for the LeNet-5 model. ^109^

**Figure 9.**
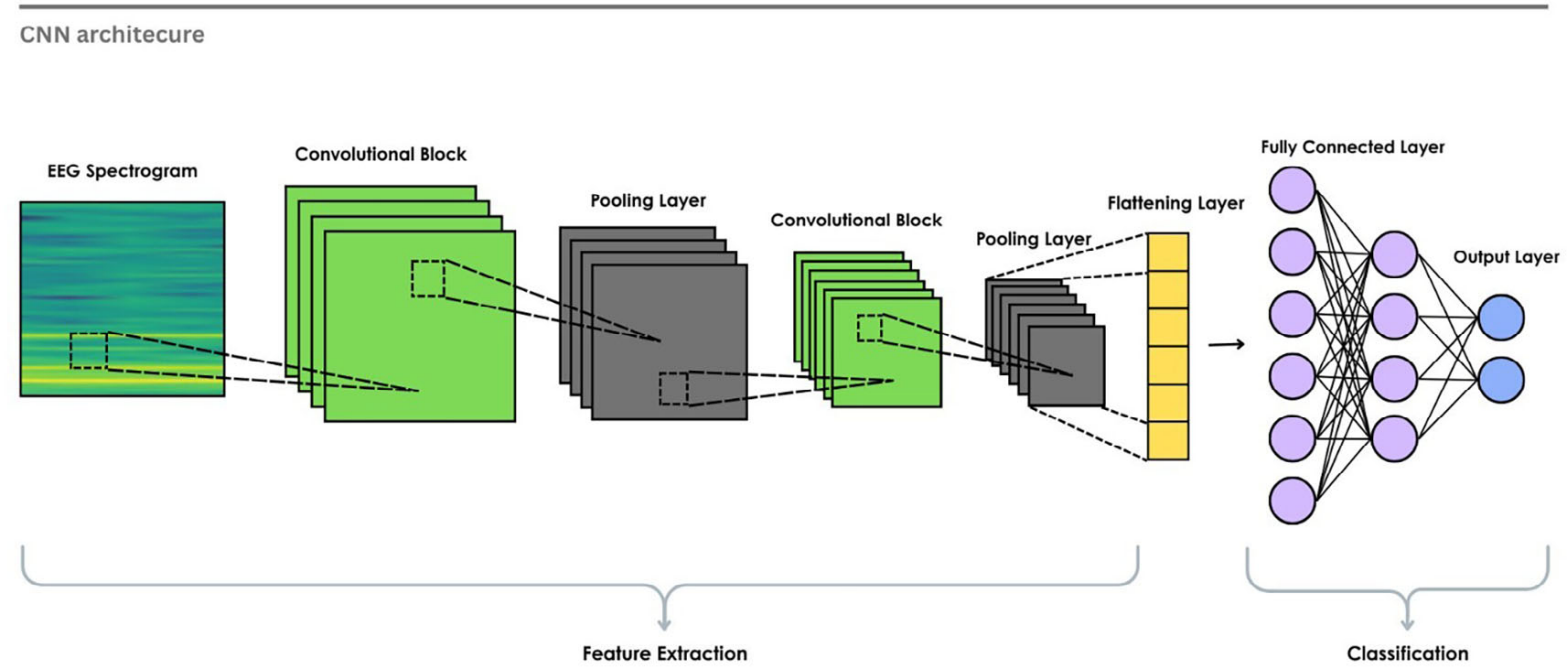
Basic CNN architecture for EEG emotion recognition.

Initially, the primary purpose of convolutional neural networks is to process grid-like data, such as images or 2D representations. The layers apply convolution operations to extract local and hierarchical features using kernels and filters. For dimensionality reduction, pooling layers are used, followed by Fully Connected (FC) layers for classification, as in Figure 9. CNNs work well for EEG-based ER when the data is converted into a 2D format, such as spectrograms or topographic images, because they can learn meaningful spatial and spectral patterns from the signal. ^110– 114^ However, despite this effectiveness, certain limitations are also observed. These studies highlight the need for a rich EEG dataset and a better channel optimization strategy. They employ data augmentation techniques, and the model’s generalization across subjects is also challenging due to inter-subject variability.

To address these limitations, further improvements were introduced. Large models such as ResNet, ^115, 116^ VGGNet, ^49^ pre-trained on various image datasets, are also used via TL ^117^ to enhance the feature extraction process from the EEG data. The process of fine-tuning a previously trained model to solve a new problem is known as transfer learning. Primarily, in the medical field, it is costly and difficult to obtain a labeled high-quality dataset. Hence, transfer learning is used to solve this issue. ^118^ Recent studies explored multimodal fusion by combining features from multiple modalities or decisions of multiple modals for improving classification result. ^119^ Lian et al. ^37^ proposed a method for combining EEG and face images, highlighting the need for real-time emotion recognition using multiple modalities. The VGGNet-16 is used as the emotion recognition model for facial images. ^120^ The joint face-EEG model obtains more than 90% accuracy on the valence and arousal dimensions of the MAHNOB-HCI dataset. It has been mentioned that the process is completed in the offline state, and to bring it to a real-world application, it is necessary to use diverse datasets and increase the emotional categories to obtain convincing results. In addition, it suggests the inclusion of the attention mechanism to process the cortical regions of interest.

**4.2.2 Recurrent Neural Networks (RNN)**

**Figure 10.**
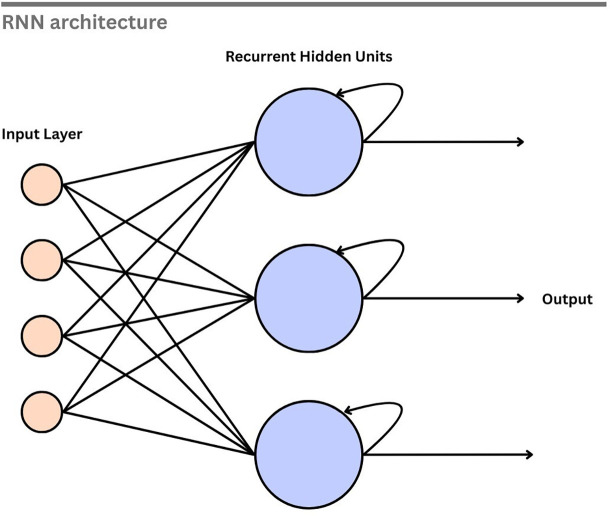
Recurrent neural network.

RNNs were proposed in the 1980s and are designed to process sequential data. RNN has a hidden state, unlike feedforward networks, which can capture information from previous steps. ^48^ Figure 10 illustrates the basic structure of an RNN. In this architecture, the input layer feeds into the hidden neurons, and each hidden neuron is linked back to itself through a recurrent connection. The information from the previous step is retained, enabling the processing of sequential data. This makes the RNN suitable for extracting the temporal patterns. By processing sequential EEG data, an RNN can capture the temporal dynamics of the signal, which is crucial for identifying the emotional state.

However, real-time processing of the EEG scalograms is a complex and computationally expensive procedure. ^121^ The Attention-based Convolutional RNN (ACRNN) model, introduced by Tao et al. ^122^ uses a CNN to extract spatial information from encoded EEG signals. It also integrates an extended self-attention mechanism on the RNN to capture more discriminative temporal features in subject-dependent experiments, and applies a channel-wise attention mechanism to adaptively weight different channels. A study uses a dual RNN for the temporal feature extraction. ^123^ However, the vanishing gradient issue with basic RNN networks during backpropagation results in a very small gradient, which stops training or takes a long time, which limits the model’s ability to learn long-term dependencies. ^59^ To overcome this issue, more advanced architectures such as LSTM and GRU are used.

**4.2.3 Long Short-Term Memory (LSTM)**

**Figure 11.**
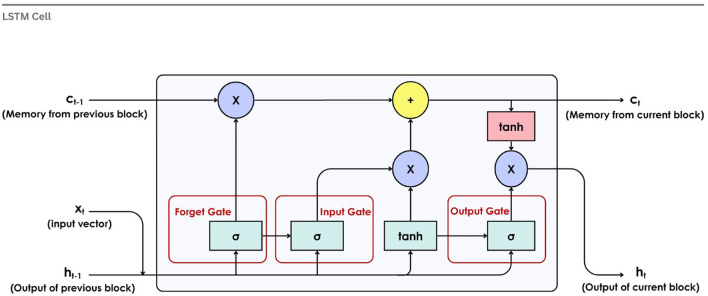
Long short-term memory cell.

In order to solve the issue of vanishing gradients in conventional RNNs, Hochreiter and Schmidhuber ^124^ introduced LSTM. This includes memory cells and gating mechanisms for learning long-range dependencies. LSTM has input, forget, and output gates to regulate the flow and retain information for very long periods, making it suitable for analyzing sequential data such as EEG signals. ^92^ Figure 11 illustrates the internal structure of an LSTM cell. At each step, the *forget gate*( *σ*) determines how much of the previous memory to retain. The new memory, *c _t_* is the outcome of the *input gate* ( *σ*) and *the tanh* function. *Output gate*( *σ*) controls how much of this new information is exposed, determining the new hidden state *h _t_.*

LSTMs might not be able to fully capture the spatial correlations between EEG channels or may be sensitive to irrelevant features and subject variability. A study used an attention mechanism-based LSTM model to select the appropriate electrodes for emotion classification. ^125^ The results have demonstrated the lobes that correspond to the valence and arousal dimensions. The domain discriminator was designed to learn features that remain consistent across different subjects. However, in subject-independent experiments, variations in data distribution between individuals often reduced performance. To overcome this challenge, researchers combined attention mechanisms with LSTMs, allowing the model to focus on the most emotion-relevant EEG channels and time segments. At the same time, domain discriminators were employed to extract features that generalize better across participants and recording sessions. ^62^

Oka et al. ^126^ proposed an LSTM-based model enhanced with an attention mechanism and optimized using Particle Swarm Optimization (PSO). While attention helps in improving the extraction of emotion-relevant features and PSO is used to optimize the hyperparameter of the LSTM network. LSTM layers were used for feature extraction in another study, which observed that they can be prone to overfitting when working with smaller datasets. ^127^ But some other research has shown that, with careful application, LSTM networks can significantly enhance the feature extraction process in EEG signals by successfully capturing long-term temporal dependencies that conventional techniques frequently fail to identify. ^128, 129^ As they model both the spatial and temporal features, hybrid models that combine LSTM with CNNs or Graph Convolutional Networks (GCNs) enhance performance by better simulating the brain’s spatial topology and temporal evolution. ^130^ Often, the temporal features of an EEG input extracted by LSTM are fused with the spatial features extracted by CNN to get a spatial-temporal feature representation, resulting in better classification performance. ^131^ Yin et al. ^30^ proposed a fusion model of LSTM and Graph CNN (GCNN), where multiple GCNN modules extract the graph-domain features, and an LSTM layer captures the temporal dynamics from the DE feature cubes. Binary classification on the DEAP dataset achieved better results, suggesting an expansion to multi-class classification. Since these developments have resulted in major improvements in classification robustness and accuracy, LSTM-based architectures are now an essential component of EEG-based emotion recognition research.

**4.2.4 Bidirectional Long Short-Term Memory (BiLSTM)**

**Figure 12.**
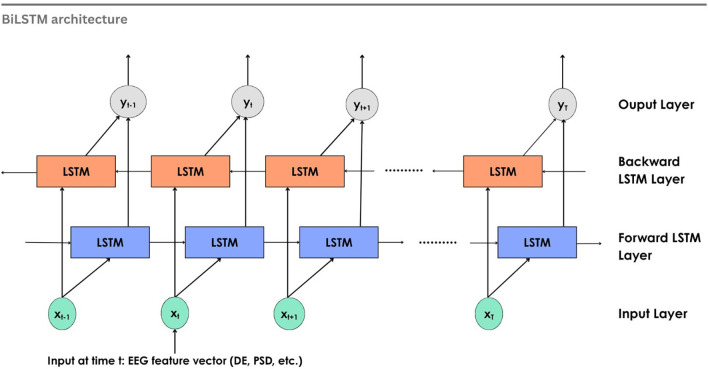
BiLSTM for EEG emotion recognition.

Schuster and Paliwal ^132^ proposed the Bidirectional RNN, which processes data in forward and backward directions using two hidden layers. The BiLSTM is built on this architecture, extending the capabilities of traditional LSTM. Two LSTM networks working in both directions allow access to the past and the future of each time point in the data sequence. The output from both the LSTMs is merged before passing on to the next layer. Emotions that are influenced by subsequent brain activity are captured by the BiLSTM network. Figure 12 shows the working of a BiLSTM network for EEG emotion recognition. At each step *t*, the input vector *x _t_* (features such as DE, PSD, etc.) is fed into the two LSTMs, one processing the input forward and another in the backward direction. The output *y _t_* is obtained by concatenating the results from both LSTMs, which can then be fed into a neural network or classifier for classification of emotions.

A study adopted BiLSTM to learn spatial-temporal characteristics within and between brain regions, highlighting that conventional LSTM might be insufficient for capturing the full context from EEG features. ^133^ The development of BiLSTM illustrates the critical importance of enriching contextual understanding in sequential data processing. A study used a hybrid model of BiLSTM and IRNN where the model is trained on the DEAP dataset with 97.19% testing accuracy and tested on the DOSE dataset, achieving 96.29% accuracy. Still, the hybrid model encountered overfitting issues. ^134^ A study performed cross-dataset emotion classification using different models and features. Two benchmark datasets, SEED and DEAP, along with a self-constructed dataset, IDEA, were also considered. The linear-formulation of DE features (LF-DE) along with the BiLSTM model was found to be performing better compared to other models and features for the same set of input. It was one of the few studies to perform cross-dataset emotional classification, and it was found that the accuracy of the classification improved compared to the existing results. But the study comes to the conclusion that the sizes of the datasets used vary and are insufficient, and this restricts the model’s performance. ^135^

Despite these promising results, BiLSTMs alone can be computationally intensive and may not fully utilize the spatial features or handle noise and variability in the EEG data. To address these limitations, researchers have established simpler hybrid models combining BiLSTM with CNNs for spatial feature extraction and resulted in robust and efficient emotion recognition. ^136^ Taking it further, the EWT-3D-CNN-BiLSTM-GRU-AT model applies Empirical Wavelet Transform (EWT) to decompose EEG signals, extracts features, and generates 3D-EEG images capturing spatial, spectral, and temporal information. A 3D CNN learns spatial features, followed by BiLSTM and GRU layers for temporal modeling, and a self-attention mechanism emphasizes emotion-relevant features. The model achieved over 90% accuracy on the DEAP dataset. ^137^

**4.2.5 Gated Recurrent Unit (GRU)**

**Figure 13.**
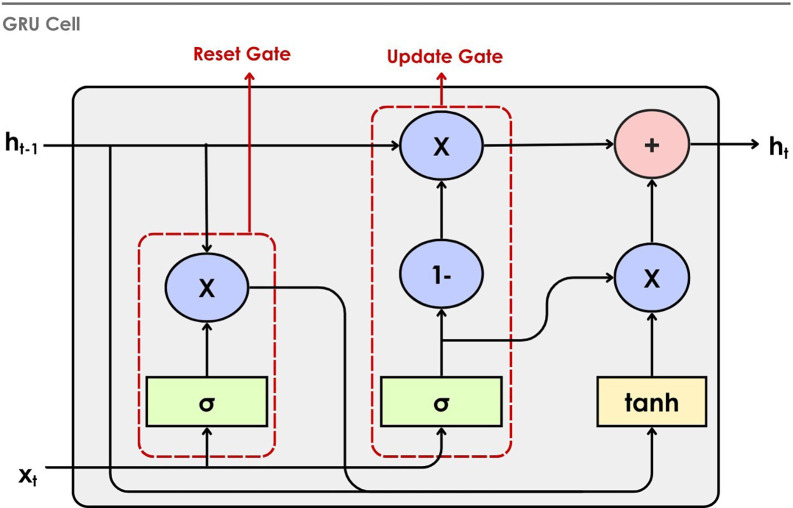
Gated recurrent unit cell.

GRU was proposed by Cho et al. ^138^ as an effective and simple gating mechanism within RNN. GRU is a simplified version of the LSTM architecture, retaining its ability to capture long-term dependencies. It relies on a reset gate to regulate the data to be forgotten and combines the input and forget gates of an LSTM into a single update gate. GRU is computationally more efficient than LSTM with fewer parameters. In the EEG environment, it strikes a balance between performance and complexity. It learns relevant time-series patterns from the data. This push toward simplicity is very important for lowering the cost of computing, speeding up training, and letting more models be used, especially in places with limited resources. The working of the GRU cell is illustrated in Figure 13. The previous hidden state, *h*_*t*−1_, and current input *x _t_* are received as input to the cell. The reset gate controls the extent to which information from the previous hidden state is discarded. After this, the input is combined with the reset-modified memory and passed through the tanh activation to generate a candidate hidden state. Finally, the update gate merges this candidate with the previous hidden state to form the new hidden state, h _t_.

A study proposed by Cui et al. ^139^ introduces a cross-subject emotion recognition method using GRU and Minimum Class Confusion (GRU-MCC). They use GRU to extract the spatial dependence of the EEG electrodes. The model achieves better accuracy by minimizing the overlap between classes. However, the need for extensive labeled data for adjusting the confusion loss and scalability to unseen datasets is an issue. In a study, GRU is used to calculate the high-level time-domain features for the SSTD model, conducting a subject-independent experiment. ^140^ One study combines convolution layers with GRU to capture spatial-spectral features along with temporal features, as GRU does not inherently capture features on the spatial and spectral level. ^141^ Houssein et al. ^142^ used BiGRU to capture temporal features. This study suggested using datasets that better simulate’real-life’ experiences, such as those that utilize video games as an emotion elicitation technique. Table 2 (refer to extended data) summarizes the supervised learning ^143– 148, 151, 153– 177, 179– 192, 194– 203, 205– 209, 211– 214, 216– 228, 231, 233– 250^ studies reported in the past five years.

### 4.3 Unsupervised DNNs

**Figure 14.**
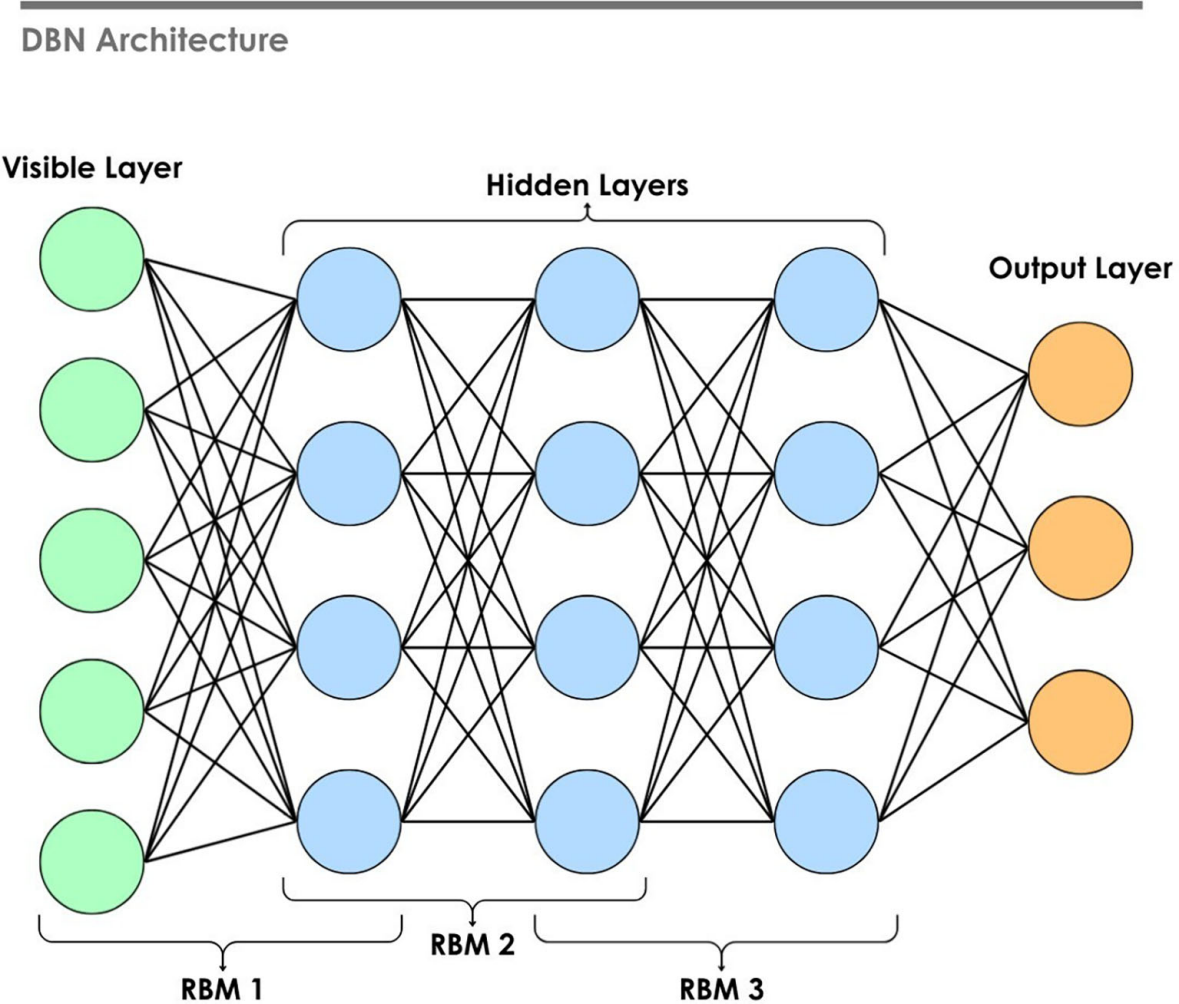
Deep belief network architecture.

Unlike supervised methods, unsupervised learning does not rely on labeled data. Objects are identified by groupings within data using methods such as clustering and dimensionality reduction. It also supports generative modeling that can be utilized for data augmentation. ^251^ Deep unsupervised methods include architectures such as Autoencoders (AEs), Generative Adversarial Networks (GANs), Restricted Boltzmann Machines (RBMs), and DBNs. Meaningful representations from complex datasets can be extracted using these methods. RBMs are generative neural networks designed for learning the probability distribution of input data, composed of visible and hidden layers with no intra-layer connections. RBMs are trained using contrastive divergence to capture hidden patterns in the data. DBNs are formed by stacking RBMs, as shown in Figure 14, and fine-tuning the resulting network with backpropagation. ^252, 253^ Hinton et al. ^254^ proposed the layer-by-layer efficient training, making it feasible to train deep networks with millions of parameters. Later, the model can be fine-tuned on the labeled data. In EEG emotion recognition, DBN can extract features from the raw or preprocessed data without the label. A study used DBN as a baseline to compare with the proposed model, Sparse Dynamic GCNN (DGCNN), which demonstrated superior performance against DBN. ^193^

In the context of EEG-based ER, concepts such as domain adaptation and transfer learning are valuable because they enable models trained on a set of subjects to generalize to new users. ^255^ This approach allows knowledge learned in a well-annotated source domain to be applied to less-labeled or unlabeled target domains, effectively bridging distributional gaps between individuals. Domain Adversarial Neural Networks (DANN) show promising results in cross-subject and cross-dataset EEG emotion recognition tasks, handling the poor generalization of emotion classification models. ^256^ The domain discriminator network encourages the system to learn domain-invariant features by focusing on emotion-relevant features. Li et al. ^116^ proposed a meta-transfer learning strategy using multi-scale features resulting in 71.29% and 71.92% accuracy for valence and arousal on DEAP and 87.05% cross-subject accuracy in SEED datasets. The meta-trained Multi-Scale Residual Network (MSRN) model is fine-tuned on a small number of labeled samples from the target subject for quickly adapting to subject-specific connectivity patterns.

**4.3.1 Generative Adversarial Network (GAN)**

**Figure 15.**
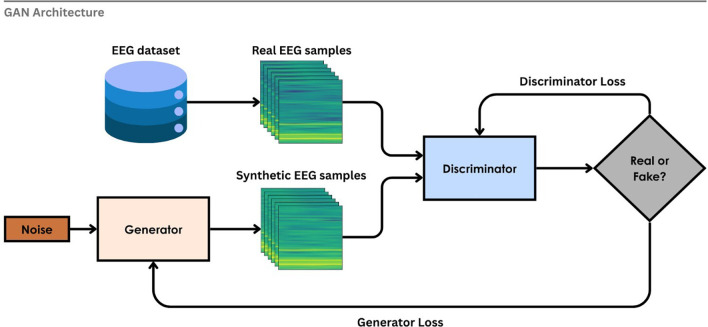
Generative Adversarial Network (GAN).

GAN was proposed by Goodfellow et al. ^257^ which uses two neural networks that are trained in opposition to one another. GAN is composed of two networks: a generator, which creates synthetic data, and a discriminator, which distinguishes between real and generated data. ^251^ This ‘game’ between these two networks enables the creation of realistic and diverse samples. To overcome the problem of limited labeled data in EEG-based emotion recognition, GAN can be used as a Data Augmentation (DA) tool that favours the real emotional patterns. ^258^ Figure 15 illustrates the working GAN for the generation of synthetic data for EEG spectrograms. Random noise is fed into the generator network, which creates artificial EEG spectrograms that are then sent to the discriminator. Real EEG samples from the training set were also sent to the discriminator at the same time. It is then backpropagated to the generator to update the parameters after the discriminator classifies it as “real” or “fake” along with the discriminator loss and generator loss. Until the discriminator fails to differentiate between synthetic and real samples, this adversarial training process is continued. ^259^

To improve the robustness of the proposed Graph Neural Network (GNN), a study utilized GAN-based domain adaptation to augment the dataset. ^260^ Gu et al. ^261^ proposed a GAN to generate EEG signal representations and combined GCNN and LSTM to identify emotions. However, it has a limitation of modal collapse that most of the GAN-related studies have, where the generator produces similar inputs without capturing the diversity of the data distribution.

**4.3.2 Autoencoder (AE)**

**Figure 16.**
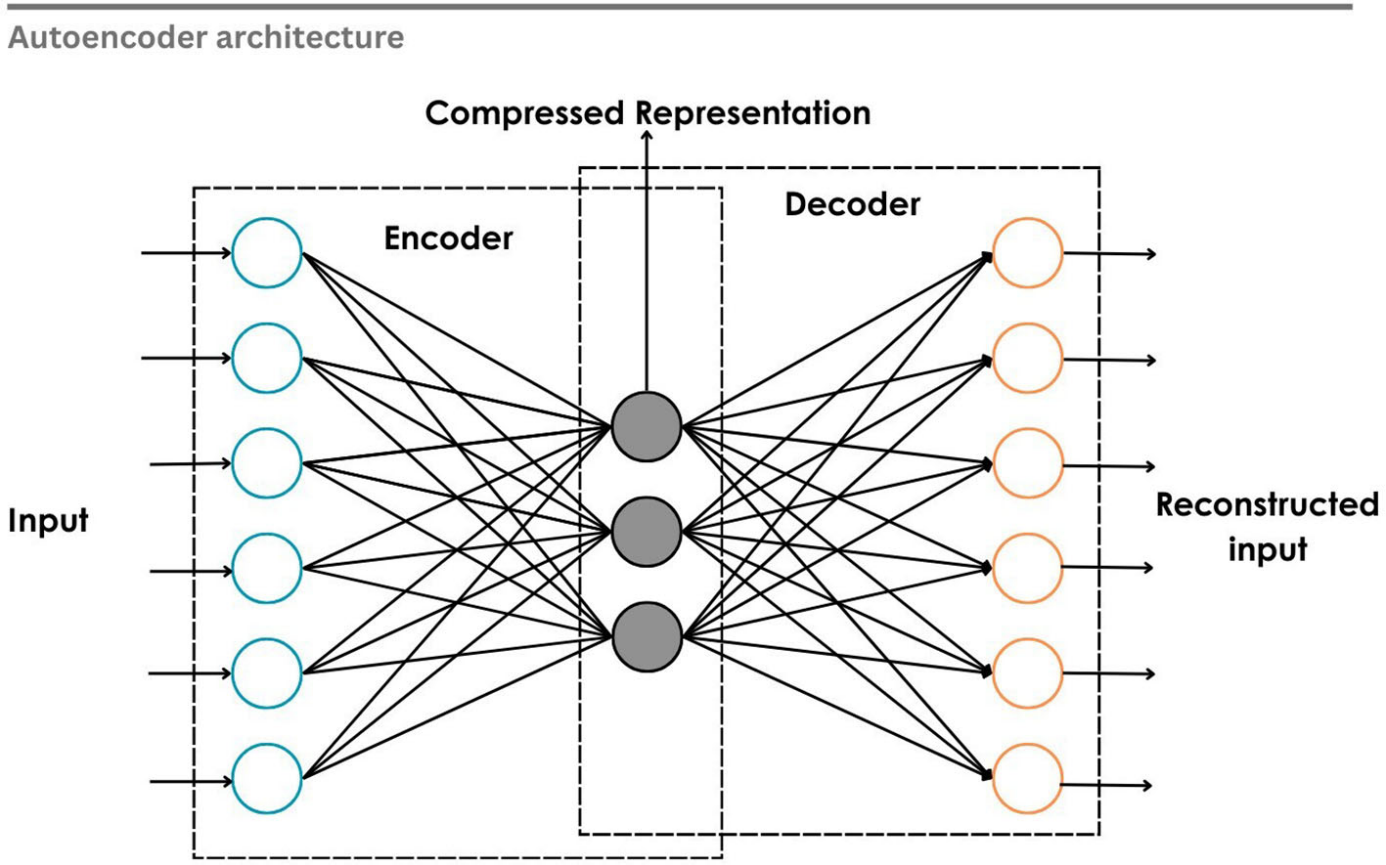
Auto encoder architecture.

Rumelhart et al. ^262^ proposed conventional autoencoders as a type of associative network that learns internal representations by back-propagating errors. It demonstrated the meaningful compressed representation of the input without the support of labels, marking a paradigm shift towards unsupervised learning. ^263^ Autoencoder has an encoder and a decoder. The encoder maps the input data into a lower-dimensional latent space, while the decoder reconstructs the original input from this compressed representation by minimizing reconstruction error, as shown in Figure 16. The autoencoder will learn meaningful features from the input data through reconstruction. Variational Autoencoders (VAE), Sparse Autoencoders (SAE), and Denoising Autoencoders (DAE) are some of the varieties. In the EEG environment, they are used for feature extraction and dimensionality reduction. Irrelevant noise in the signal can be filtered out while capturing the most important features. The latent representation can be given as input to a classifier or can be clustered.

Wang et al. ^264^ uses a Multi-Modal Domain Adaptive VAE (MMDA-VAE) to provide a combined representation of multiple modalities for enhancing cross-domain emotional classification on SEED and SEED-IV. In this study, a VAE is used to project data from various modalities onto a single latent space. While the model achieves improved performance, its limitations are also noted. The encoder and decoder in VAE produce a large number of parameters. The method assigns the same weight to the two modalities. So, it is suggested to use an adaptive weighting mechanism in the upcoming studies.

It is also advised to use additional physiological signals such as GSR, EMG, and ECG. Li et al. ^265^ uses a VAE for learning the spatial-temporal latent features. The class imbalance problem in the dataset was also highlighted in this study. Liu et al. ^102^ used a CNN-SAE-DNN model for emotion classification, where CNN and SAE are used for feature extraction and DNN for the final classification. In order to improve performance, the author recommends that different AE designs, including stacked or variational autoencoders, be investigated in the future. It is also pointed out that the label information was used in the feature extraction, which raises questions about biases and overfitting. Pang et al. ^266^ proposed the Multi-Scale Masked Autoencoders (MSMAE) model, which is trained on a large scale of unlabeled EEG data to extract subject-invariant features. These features are then fine-tuned on a small amount of labeled data from a specific subject for personalization. An attention feature extractor, *Attn*, is used for aligning features of pre-training and fine-tuning data. But the current performance of the MSMAE model relies on the handcrafted features, resulting in information loss. An overview of recent unsupervised methods ^267– 288^ from the last five years is presented in Table 3 (refer to extended data).

### 4.4 Hybrid learning methods

Hybrid learning is a combination of supervised and unsupervised methods. Supervised models perform better on labeled data but cannot learn from unlabeled data. Generative models are flexible because they can learn from both labeled and unlabeled data. The hybrid method combines both approaches to benefit from their individual strengths. These semi-supervised methods can benefit from small labeled datasets while utilizing a large amount of unlabeled data to find patterns and increase robustness. Hybrid models can be a combination of (1) different supervised or unsupervised methods (e.g., CNN+RNN, GAN+AE) or (2) a supervised and an unsupervised model (e.g., AE+CNN, GAN+GCN).

Integrating CNN and RNNs has become common in EEG-based emotion recognition because CNNs extract spatial and spectral features from EEG input, while RNNs like LSTM or GRU extract temporal dynamics. Zhang et al. ^283^ uses a stacked depthwise separable CNN for extracting spatial and spectral features and an LSTM for temporal information, incorporating multiple attention mechanisms. A 4D feature representation is constructed of DE and Absolute Power (AP) features from four frequency bands. However, the feature representation is weaker due to the use of 2D mapping matrices for representing spatial relationships among electrodes. MobileNet RNN (MRNN) proposed in uses a pre-trained MobileNet model together with an RNN for spatial and temporal feature extraction, respectively. ^121^ While it captures EEG dependencies effectively, it poses challenges in real-time applications due to computational load. The limited data scale also restricts improvements in deep neural network-based emotion recognition. Li et al. ^284^ proposed Spiking Spatiotemporal Neural Architecture Search (SSTNAS), combining spiking CNN for spatial and a spiking LSTM for temporal feature extraction. The study used the XAI tool Shapley Additive exPlanations (SHAP) for model interpretability and pointed out the high computational cost and poor cross-dataset performance. The need for lightweight models and domain generalization is emphasized.

Generative models are widely used in hybrid approaches. In a study, an Extreme Learning Machine Wavelet Autoencoder (ELM-W-AE) was used for data augmentation. ^285^ Even after ResNet-18 was used for classification with 99.6% accuracy, the study fails to explain the model’s generalization ability. Mohajelin et al. ^286^ and Gilakjani and Osman ^260^ use GANs for augmenting the dataset and Graph Neural Networks (GNNs) for classification, highlighting the limited emotional categories present in current databases. A study used a VAE-GAN model for generating high-quality artificial samples by segmenting DE features temporally and spatially. ^287^ However, the network has a complex structure and relatively high time complexity. An unsupervised VAE was used to learn spatio-temporal latent features without labels in a study. ^265^ A parallel branch with GCN and GRU extracted spatio-spectral features from labeled data. The representations from both branches were fused to obtain subject-independent emotion classification. The model employed a multi-task training strategy, increasing computational demand and complexity due to the dual branches. Zhang et al. ^106^ proposed a self-supervised data augmentation framework using a masking reconstruction GAN that reconstructs masked portions of EEG signals. The synthesized EEG samples are then used along with real EEG data to fine-tune a supervised classifier. According to the authors, the current classifier, STNet, is not designed to handle distribution shift across subjects.

Mai et al. ^288^ proposes a wearable EEG system focused on EEG signals from the ear rather than traditional scalp EEG devices. The system uses a superlets-based signal-to-image conversion framework, which transforms EEG signals into 2D-images, making it convenient to process using a modified ViT integrated with Shifted Patch Tokenization (SPT) and Locality Self-Attention (LSA). The model achieved an accuracy of 92.39% on the self-collected dataset, outperforming ResNet-50 and EfficientNet-B0 models. Even though the model offers limited spatial coverage causing a potential data loss. In the DRS-Net model, ^289^ a dynamic reservoir-state encoder is employed to capture spatio-temporal features from multi-channel EEG data. The extracted features are then processed through an LSTM-dense decoder for emotional state classification. The reservoir computing processes the temporal sequences efficiently, and LSTM handles long-term dependencies. The model performance drops on subject-independent experiments. Zhang et al., ^290^ proposed a Python toolbox built on PyTorch, named TorchEEG *_EMO_*, where the workflow is divided into five distinct modules (dataset, transforms, model selection, models, and trainers) to provide plug-and-play functionalities. Also, a window-centric input-output system is introduced. The model provides almost all popular EEG datasets, preprocessing techniques, state-of-the-art EEG models, and evaluation strategies. In a study, a Semi-skipping-Layered Gated Unit (SLGU) was used to automatically skip the divergent factor during the network training. ^291^ SLGU-ENet was used for deep feature extraction and Support Vector Networks (SVN), Naive Bayes (NB), and KNN were used for classification. In order to reduce the computational cost a reduction function named bag of visualized characters (BoVC) is used.

Wang et al. ^292^ proposed a knowledge distillation-based model, where a large transformer-based teacher model was trained on labeled source EEG samples. A lightweight BiLSTM student model mimics the teacher’s feature representation and is further refined using Domain Adversarial Neural Network (DANN) by aligning features between labeled source and unlabeled target domains. The model’s performance depends on the generalization ability of the teacher and it is computationally expensive than single stage method. The DC-ASTGCN model combines a Deep CNN (DCNN) with an Adaptive Spatiotemporal GCN (ASTGCN) enhanced with an attention mechanism and adaptive modules, enabling the extraction of both local frequency-domain features and spatio-temporal connectivity patterns from EEG signals. ^293^ Hybrid Model with Improved Feature set for Emotion Detection (HMIFED) employs a hybrid architecture that combines BiLSTM and an improved RNN (IRNN) for cross-dataset emotion recognition. ^134^ Although the model achieves good accuracy, it still encounters overfitting issues and lacks generalization. CIT-EmotionNet consists of a CNN and Transformer network module processed simultaneously. The CNN captures local image features, while the Transformer captures global dependencies. ^294^ CIT-EmotionNet achieved a maximum average recognition accuracy of 98.57% on the SEED dataset and 92.09% on the SEED-IV dataset. Table 4 (refer to extended data) provides a detailed summary of hybrid approaches ^295– 335, 337– 343^ investigated over the last five years.

Graph Convolution Networks (GCN) are used in emotion recognition because they can process non-Euclidean and graph-like structures of EEG data, capturing the spatial relationships between EEG electrodes very well. Song et al. ^232^ proposed the Graph-Embedded CNN (GECNN) model, combining a CNN and a graph module for extracting both local and global features. Both subject-dependent and independent operations were performed on SEED, SDEA, DREAMER, and MPED datasets. There is a gradual drop in the performance of the model for subject-independent evaluation. Since the model focuses on the local and global spatial features, temporal information is rarely considered. An Ordinary Differential Equation (ODE)-based GCN was proposed by Chen Y et al. ^129^ combining ODE-driven spatial propagation and Dynamic Time Wrapping (DTW)-based temporal alignment for improved valence/arousal classification. However, the proposed method assumes smooth and continuous EEG signal with stable emotional transitions according to “homophily” assumption, making it less effective when abrupt signal or emotion fluctuation occur. The hierarchical dynamic GCN proposed in a study uses a gated graph convolution to capture both intra- and inter-region dependencies. ^210^ A subject-independent experiment on the SEED dataset provides 89.23% accuracy. Siam-GCAN introduces a twin-branch GCN with shared weights to process pairs of EEG trials. Both branches apply the same graph convolutions over the electrode adjacency graph, followed by a multihead attention mechanism. ^230^ Recently, models based on Capsule Networks (CapsNet) have shown strong performance efficiency in emotion recognition, addressing challenges like cross-subject variability, spatial feature extraction, and channel redundancy. ^344^ In their work, Wei et al. ^331^ developed TC-Net, which leverages a Transformer module to extract global features and an Emotion Capsule module to model spatial features among EEG channels, achieving good results (≈98%) on both DEAP and DREAMER datasets. The study focused only on the subject-dependent scenario. The DA-CapsNet model introduced a multi-branch CapsNet, where each branch processes DE features from different frequency bands. ^273^ By integrating domain adaptation, the model significantly improves cross-subject emotion recognition across DEAP, DREAMER, and SEED datasets. The model is highly dependent on the quality of domain adaptation. Another model, Bi-CapsNet, employs binary weights and activations to lower computational cost and memory usage while still maintaining strong accuracy, making it suitable for deployment on mobile devices. ^204^ It achieves 25× reduced computational complexity and 5× less memory usage, while only *less than* 1% drop in accuracy. The accuracy of the model is highly reduced when directly used in the subject-independent scenario. The Hierarchical Attention-CapsNet(HA-CapsNet) model integrates 3DCNN and CapsNet with a hierarchical attention mechanism (local and global attention), extracting rich spatio-temporal data from multidimensional EEG data. ^53^ The generalization of the model to more diverse, real-world data is not available. Jana et al. ^229^ proposed a method where sparse spatio-temporal frames are created from EEG data, combining the position of electrodes and temporal dynamics. Capsule neural dynamics are utilized to model both local and global spatial-temporal information. The accuracy of the model seems to be reducing during the cross-subject experiment, showing the difficulties in generalizing the model to unseen subjects. Several other studies also use capsule networks as spatial features extractors and classifier achieving better classification results all over. ^96, 150, 215, 301^

Concepts such as XAI or uncertainty analysis are important for a DL-based model because they make it more interpretable, trustworthy, and relevant. ^63^ XAI helps to reveal how and why a deep learning model predicted that specific emotion based on the given EEG signal. To build trust in the results of affective computing and BCI systems, interpretability is important. XAI will help in highlighting the regions or frequencies of the brain that contribute to the result, which will improve the clinical explanation of the model. Similarly, uncertainty analysis quantifies the confidence the model has in its predictions. ^345^

EEG-ConvNet proposed by Khan et al. ^111^ used the XAI techniques Gradient Class Activation Mapping (Grad-CAM) ^346^ and Integrated Gradients (IG) to interpret the predictions. The bagging ensemble approach is utilized for robustness, and the IG method is employed on the ResNet-34 model. Chaudary et al. ^152^ proposed the souping of EEG-CNN models trained on EEG scalograms of different sizes. It also incorporates Grad-CAM visualization for the interpretability of the model. Chen et al. ^347^ used uncertainty to guide the augmentation of EEG graph data. Uncertainty is quantified and utilized to adaptively augment graph connectivity. It enhances the module’s ability to handle distribution shifts in unseen subjects. In the model Connectivity Uncertainty GCN (CU-GCN), uncertainty is used to represent the spatial dependencies and temporal-spectral relationships in EEG signals, guiding the construction of the adjacency matrix through a Bayesian framework. ^178^

### 4.5 Multimodal emotion recognition

This review is mostly focused on EEG-based unimodal emotion recognition, but it’s important to note that multimodal techniques that combine other physiological and behavioural data have made a lot of progress. Multimodal emotion recognition models are often developed by pairing EEG with behavioural or physiological modalities (like facial expression or GSR) to provide a richer understanding of emotion responses. These models can usually perform better than unimodal systems in cross-subject emotion classification tasks because the brain-level signals (EEG) can provide clearer representations of emotional states. ^348^ Cui et al. ^349^ proposed a multimodal approach combining EEG and facial expression, using a cross-modal attention that fuses the EEG vector with a ConvLSTM (with spatial-attention) face encoder for classification. The multimodal approach provides better results than using a single modality (face or EEG). Using the MAHNOB-HCI data set, Sedehi et al. ^350^ examined the causal relationship between pupil dilation levels, EEG and ECG. Granger-causality maps are used to analyze causation, and the ResNet-18 model is used for classification. ^351^ Irrelevant frames are discarded to enhance the signal quality. But the method is limited to only two emotional classes. Pan et al. ^352^ propose Deep-Emotion, a multi-branch architecture that fuses EEG, facial, and speech inputs using GhostNet, Lightweight Full CNN (LFCNN), and tree-like LSTM (tLSTM), respectively. A decision-level fusion method was used to combine the results from multiple modalities, resulting in a more accurate and collective result. The authors question the generalizability of the model using the public datasets in the real-time scenario. Li et al. ^353^ proposed a Deep EEG-first Multi-physiological Affect (DEMA) that combined EEG with other physiological signals such as blood pressure, GSR, respiration belt, and temperature. The model introduced the Affective Influence Matrix (AIM) to align and unify multimodal representation by assessing the influence of EEG on the modalities. Fu et al. ^32^ proposed a multimodal approach combining EEG with eye movement signals, where a feature guidance module is used to direct the extraction of eye movement features. The objectivity of emotion estimation and individual differences are two of the problems highlighted.

In conclusion, recent multimodal emotion recognition literature has repeatedly demonstrated that emotion classification performance and real-world robustness will be improved through the integration of EEG data with other physiological or behavioural signals (facial expression, speech, ECG, and eye movement). Today, many of the most recent state-of-the-art DL methods that use cross-modal attention, transformers, and domain adaptation have made the process of combining these independent types of data even more effective. The combination of the complementary features of the different modalities by deep learning will help to provide users with advanced emotional recognition systems that offer even less biased and more reliable emotional recognition. But multimodal approaches have their own challenges, including increased system complexity, the need for synchronized data collection, and the selection of an appropriate fusion strategy. Yet multimodal approaches have the capacity to aid the advancements in the field of affective computing, enabling more natural emotion computing systems and human-computer interaction.

## 5. Challenges and future directions

Due to advancements in ML and signal processing, the field of emotion recognition has advanced significantly. The usage of EEG to classify emotion has increased because EEG models the brain activity during the emotion elicitation better than any other mode of ER. The usage of neural networks for ER has transformed HCI and affective computing by offering automatic, data-driven feature extraction/fusion and efficient classification processes. DL models, including CNN, RNN, LSTM, GAN, and more recently GCN and CapsNet have been able to outperform the traditional machine learning methods by modeling spatial, temporal, and other patterns present in the EEG data, which is complex by nature.

### 5.1 Challenges

• ***Challenges in data representation:*** Several EEG emotion recognition studies have analyzed data from EEG data collected in controlled laboratory settings with passive stimuli, such as videos, images, or sounds. In contrast, emotional states in real-world situations are in fact more often dynamic, complex, and driven by some situations. The difference between EEG collected in a lab setting and emotional states in the natural environment potentially raises concerns with reliability. Models trained on experimental data may have difficulty in generalizing when it is applied to real-world situations such as classrooms, workplaces, or health care settings.
• ***Challenges in emotion labels:*** Another important issue is the small set of emotion labels that are employed in the majority of studies. Most benchmark datasets are limited to the basic emotional dimensions—usually valence, arousal, or a few discrete categories, such as happiness, sadness, or anger. Even if these results make for easier model training, it restricts the richness and detail of emotional understanding. Often the emotions such as stress, boredom, and frustration are not seen in any datasets.
• ***Real-world feasibility and complexity of the model:*** Many of the high-performing models mentioned in this study have complicated architectures with an increased number of parameters, causing intense computational power. It is practically difficult to deploy these models in the real world where a system for emotion recognition should have very low computational power and better recognition accuracy. Such a situation suggests a shift towards the development of lightweight, efficient, scalable architectures that perform well without losing its practicality.
• ***Generalization of the model:*** EEG signals are naturally variable between subjects resulting from psychological and cognitive differences occurring across individuals. Therefore, a model trained on a user or group of users will probably not generalize well to other user groups—this has a serious role in real world applications. This highlights the importance of developing cross-subject models that are not affected by subject-to-subject variability.
• ***Need for interpretability:*** DL models naturally act like a black box. They do not clearly explain how it came to that particular conclusion. With the increasing complexity of DL models, the interpretability of their decisions becomes increasingly challenging - an area of concern, especially for clinical or psychological applications.

### 5.2 Future directions

There are a number of promising strategies and research directions that can be used to get around the problems that are currently making EEG-based emotion recognition systems less useful and effective:

• There is a growing demand to go beyond traditional laboratory-based video clips, images, and audio-based emotion elicitation. While controlled experimental designs provide precision, they do not specifically reflect the real-life characteristics of emotion. For the field to evolve, future research should collect EEG data in more natural real-life scenarios, such as games and virtual reality, that more accurately reflect the complexities of emotional experience encountered daily. To analyze the temporal trajectory of the emotions, the emotional history of the person can be considered. This could include continuous data collection for a long period using an emotionally rich stimulus.
• To capture the full complexity of human emotion, datasets should not be limited to the typical psychological dimensions of valence and arousal but should consider emotions in a wider range of categories. This could mean using models based on multiple labels for emotions. The reliability of labeling can be increased by working with psychological researchers to define specific emotion categories. Linguistic methods such as Natural Language Processing (NLP) can also be integrated with EEG to understand the complex emotional states.
• To facilitate real-world application, such as mobile or embedded systems, the researchers need to focus on building models that are computationally efficient. Techniques such as model compression, quantization, and knowledge distillation can significantly reduce the size of a model while having minimal impact on its performance. Also, temporal evolution modeling can be followed, which predicts how emotional states evolve over a few seconds or minutes instead of classifying the snapshots of emotion. This will help in the real-time stress prevention and adaptive learning systems.
• Improving cross-subject and cross-dataset generalization is essential. Transfer learning — where knowledge learned from one dataset is adapted to another—can significantly reduce the amount of training data needed for new subjects. Domain adaptation methods, including adversarial training and domain-invariant feature extraction, can help models adapt to different users or recording conditions.
• Making use of XAI tools, such as attention visualization, feature importance mapping, or saliency mapping can increase interpretability and trust in deep models, especially in clinical or diagnostic use cases. Further, uncertainty quantification tools, such as Bayesian deep learning or Monte Carlo dropout, can be used to evaluate the confidence of predictions, increase reliability, and inform decisions based on predictions of a deep model in sensitive situations.

## 6. Conclusion

In summary, emotion recognition systems have made great advancements over the past few years with the developments in the methods using DL tools. The availability of affordable EEG devices has accelerated research on EEG-based emotion recognition in affective computing. Although technically challenging, emotion recognition in EEG-based systems is still a very promising field. Apart from the traditional datasets, latest studies tend to use the self-collected EEG dataset for getting access to more diverse signals among subjects from different regions, age groups, and health conditions. ^95, 149, 336^

In this review, a detailed study of 233 Q1-ranked journals from 2020 to 2025, collected from 5 databases using specific keywords, is performed. The public datasets used for EEG signals in emotion recognition are also analyzed, along with their preprocessing steps, emotion annotation levels, and the number of EEG channels used. The review employed the Rayyan AI tool for duplicate removal and screening of papers. The DL-based methods were further categorized into supervised, unsupervised, and hybrid approaches for better understanding. Different DL pipelines are discussed based on feature representation, deep feature extraction, and the type of evaluation methods they follow. Hierarchical, spatio-temporal features can be learned from raw EEG signals using DL techniques, particularly attention-based and hybrid models. Along with subject-dependent evaluation, subject-independent experiments are commonly conducted in EEG-based emotion recognition studies to evaluate how well a model generalizes to unseen subjects. Even so, subject-dependent approaches remain critical for developing personalized emotion recognition systems. Some researchers have started exploring semi-supervised techniques in which large amounts of unlabeled EEG data are used to train the models, then later, these models are fine-tuned on a small amount of labeled or personal data. This approach helps to optimize the trade-off between personalization and scale. Since generalization is critical in real-world scenarios, subject-independent or cross-subject evaluation is often a better indicator of a model’s robustness. Apart from that, researchers have also used self-supervised learning to learn rich EEG representations from pretext tasks without labels, followed by fine-tuning. TL and domain adaptation are increasingly used to improve performance when there’s a distribution shift across subjects or datasets.

Along with the other advances, the emergence of XAI and uncertainty modeling have made it possible to create more interpretable and reliable systems. These developments are critical for applications such as personalized interfaces, healthcare, and education, where understanding the ‘how’ behind a prediction is just as important as the prediction itself. In conclusion, EEG-based emotion recognition has presented remarkable opportunities to understand and respond to human emotion. While there has been significant progress with computational techniques and model construction, limitations with generalizing across subjects, robustness in the real world, and computational efficiency continue to persist. More research concentrating on scalable, interpretable, and adaptable techniques will be needed to overcome these challenges. Emotionally intelligent systems that can support healthcare use cases, improve individual user experience, and enable a more natural human-machine interaction are anticipated as this field advances.

## Data Availability

Mendeley Data: Deep Learning Techniques for EEG-Based Emotion Recognition: https://doi.org/10.17632/vxg52py2nw.3 ^354^ This project contains the following undelaying data.
•
PRISMA_2020_abstract_checklist.docx (PRISMA abstract checklist) •
PRISMA_2020_checklist.docx (PRISMA checklist) •
PRISMA_2020_flow_diagram.docx (PRISMA flow diagram) Data are available under the terms of the Creative Commons Zero “No rights reserved” data waiver (CC0 1.0 Public domain dedication). Mendeley Data: Deep Learning Techniques for EEG-Based Emotion Recognition: https://doi.org/10.17632/vxg52py2nw.3 ^354^ This project contains the following undelaying data.
• graph_data.xlsx (Contains the raw data for the two graphs, Figure 6 and Figure 7) • Table.xlsx (Data associated with all the tables in the article) Data are available under the terms of the Creative Commons Zero “No rights reserved” data waiver (CC0 1.0 Public domain dedication).
