# Supplementary material for: A Review of Deep Learning Techniques for EEG-Based Emotion Recognition: Models, Methods, and Datasets
Source: F1000Res. 2026 Mar 9;14:1276. Originally published 2025 Nov 18. [Version 2] doi: 10.12688/f1000research.171170.2 (PMC12825124; doi:10.12688/f1000research.171170.2)
Supplement: Peer review history

# Reviewer report: Reviewer response for version 2

**DOI:** 10.5256/f1000research.196597.r466052

**Published:** 2026-03-10

**Authors:** Dr. Kevin Noronha

**Affiliations:** 1 St. Francis Institute of Technology, Mumbai, Maharashtra, India

The authors have addressed all the queries and suggestions raised during the review process satisfactorily. The revisions made have improved the clarity and quality of the manuscript. I have no further comments to make. From my side, the paper can be accepted for indexing.

Are the rationale for, and objectives of, the Systematic Review clearly stated?

Yes

Is the statistical analysis and its interpretation appropriate?

Not applicable

If this is a Living Systematic Review, is the ‘living’ method appropriate and is the search schedule clearly defined and justified? (‘Living Systematic Review’ or a variation of this term should be included in the title.)

Not applicable

Are sufficient details of the methods and analysis provided to allow replication by others?

Yes

Are the conclusions drawn adequately supported by the results presented in the review?

Yes

Reviewer Expertise:

Biomedical Signal and Image processing

I confirm that I have read this submission and believe that I have an appropriate level of expertise to confirm that it is of an acceptable scientific standard.

# Reviewer report: Reviewer response for version 1

**DOI:** 10.5256/f1000research.188735.r436872

**Published:** 2026-01-06

**Authors:** Amjad R. Khan

**Affiliations:** 1 Prince Sultan University, Riyadh, Saudi Arabia

The review article makes a valuable contribution but requires further improvement before it can be considered for Indexing. 

 After evaluating this paper, I have minor corrections that could help improve the final version of the article. The most important are as follows:

 This artificially narrows the literature and excludes relevant works published in reputable conferences, which are common in EEG and DL domains.

 It creates a bias toward highly resourced groups and does not capture emerging methods or datasets published elsewhere.

 Many sections list models and architectures but lack deep technical critique, for example:

 CNN–RNN hybrid limitations mentioned only briefly (e.g., weak spatial mapping using 2D matrices) 

 LSTM overfitting mentioned but not analyzed in context of dataset size and augmentation quality 

 GAN/AE models discussed without evaluation of failure cases such as mode collapse or distribution drift 

 Overall, the review becomes descriptive instead of analytical.

 No unified comparison table is included for benchmark datasets.

 The paper frequently notes cross-subject generalization problems (e.g., SSTNAS poor cross-dataset performance) but does not: 

 compare domain adaptation techniques

 analyze failure modes

 summarize robustness challenges

 Finally, I can see many ref. are old and not fully related to the article theme. So I would suggest that authors should strengthen the literature review and analysis section if the authors include the following citations & compare the results in the article. (Refer below)

 Ref no 1

 Ref no 2

 Ref no 3

 Ref no 4

 Ref no 5

 Ref no 6

 Ref no 7

Are the rationale for, and objectives of, the Systematic Review clearly stated?

Yes

Is the statistical analysis and its interpretation appropriate?

Yes

If this is a Living Systematic Review, is the ‘living’ method appropriate and is the search schedule clearly defined and justified? (‘Living Systematic Review’ or a variation of this term should be included in the title.)

Partly

Are sufficient details of the methods and analysis provided to allow replication by others?

No

Are the conclusions drawn adequately supported by the results presented in the review?

Yes

Reviewer Expertise:

Medical imaging, cancer diagnosis, Deep learning

I confirm that I have read this submission and believe that I have an appropriate level of expertise to confirm that it is of an acceptable scientific standard, however I have significant reservations, as outlined above.

## References

[1] : Enhanced Feature Selection for Imbalanced Microarray Cancer Gene Classification Using Chaotic Salp Swarm Algorithm. *International Journal of Theoritical & Applied Computational Intelligence* .2025; 10.65278/IJTACI.2025.16 259-283 10.65278/IJTACI.2025.16

[2] : A comprehensive study of mobile-health based assistive technology for the healthcare of dementia and Alzheimer’s disease (AD). *Health Care Management Science* .2020;23(2) : 10.1007/s10729-019-09486-0 287-309 10.1007/s10729-019-09486-0 31218511

[3] : Feature Selection Methods in Big Medical Databases:A Comprehensive Survey. *International Journal of Theoritical & Applied Computational Intelligence* .2025; 10.65278/IJTACI.2025.21 181-209 10.65278/IJTACI.2025.21

[4] : Retinal imaging analysis based on vessel detection. *Microscopy Research and Technique* .2017;80(7) : 10.1002/jemt.22867 799-811 10.1002/jemt.22867 28294460

[5] : Detection and Classification of Skin Cancer by using CNN-Enabled Cloud Storage Data Access Control Algorithm based on Blockchain Technology. *International Journal of Theoritical & Applied Computational Intelligence* .2025; 10.65278/IJTACI.2025.31 146-159 10.65278/IJTACI.2025.31

[6] : Image Enhancement and Segmentation Techniques for Detection of Knee Joint Diseases: A Survey. *Current Medical Imaging Reviews* .2018;14(5) : 10.2174/1573405613666170912164546 704-715 10.2174/1573405613666170912164546

[7] : Cloud‐based decision support system for the detection and classification of malignant cells in breast cancer using breast cytology images. *Microscopy Research and Technique* .2019;82(6) : 10.1002/jemt.23222 775-785 10.1002/jemt.23222 30697861

# Response

**Published:** 2026-02-25

**Authors:** Raghavendra U

**Affiliations:** Instrumentation and Control En, Manipal Institute of Technology, Manipal, Karnataka, India

**Reviewer comment:** Relevant works published in reputable conferences are excluded creating a bias toward highly resourced groups.

**Response: **Thank you for this valuable comment.

 The present study includes 233 peer-reviewed articles published in Q1-ranked journals. Conference publications were initially limited to maintain a manageable scope. However, as per the suggestion of the reviewer, we have now incorporated several high-impact conference papers (References 355-359) and appropriately cited them in the revised manuscript.

**Reviewer comment:** CNN-RNN hybrid limitations are mentioned only briefly.

**Response:** We sincerely thank the reviewer for this valuable comment. In response, appropriate revisions have been made in the second paragraph of Section 4.4 of the manuscript.

**Reviewer comment:** LSTM overfitting mentioned but not analyzed in context of dataset size and augmentation quality.

**Response:** We appreciate the reviewer’s valuable feedback. Accordingly, the necessary changes have been incorporated into the Section 4.2.3 in the revised manuscript.

**Reviewer comment:** GAN/AE models discussed without evaluation of failure cases such as mode collapse or distribution drift.

**Response: **We sincerely thank the reviewer for this valuable comment. The failure cases of the generative models have now been incorporated into Paragraph 4 of Section 4.3.2 in the revised manuscript.

**Reviewer comment:** No unified comparison table is included for benchmark datasets.

**Response: **The detailed dataset characterization has been provided in the Extended Data file as instructed by the journal editors, where a comprehensive comparison of the datasets is presented (refer to Table 1).

**Reviewer comment:** The paper does not compare domain adaptation techniques.

**Response: ** We sincerely thank the reviewer for this valuable comment. A comparison of different domain adaptation approaches, along with their failure modes, has now been discussed in the last two paragraphs of Section 4.3.2, supported by representative studies from the literature.

**Reviewer comment:** The paper does not summarize robustness challenges.

**Response: ** Thank you for this valuable comment. In response, the discussion on robustness-related challenges has been added to Section 5.1 of the manuscript.

**Reviewer comment:** Many references are old and not fully related to the article theme. So, I would suggest that authors should strengthen the literature review and analysis section if the authors include the given citations (7 references) and compare the results in the article.

**Response:** We sincerely thank the reviewer for this valuable insight. The earlier references were primarily included to introduce the foundational concepts of emotions, affective computing, and machine learning. In line with the reviewer’s suggestion, we have strengthened the literature review by incorporating a broad range of recent and relevant studies for the up-to-date coverage of the field.

# Reviewer report: Reviewer response for version 1

**DOI:** 10.5256/f1000research.188735.r434558

**Published:** 2025-12-30

**Authors:** Dr. Kevin Noronha

**Affiliations:** 1 St. Francis Institute of Technology, Mumbai, Maharashtra, India

The manuscript presents a systematic review of deep learning (DL)-based emotion recognition (ER) using electroencephalography (EEG). The review is based on the PRISMA guidelines. **T**he authors surveyed five major scientific databases to identify the research work published between 2020 and 2025. They have shortlisted 233 studies after the screening.

 The focus of the article is mainly to review the Deep learning architectures and methodological choices used in EEG-based emotion recognition. Authors have also done a survey on publicly available EEG–ER datasets and analysed their experimental paradigms, emotional labelling frameworks, and elicitation protocols. They have also tried to highlight the evolution of interpretability, generalizability, and data-efficiency in DL models for EEG. At the end they have provided future research recommendations toward building reliable, scalable EEG-based ER systems.

 The review claims to offer a roadmap for upcoming research and to identify current methodological bottlenecks. The topic is important and timely, and a systematic synthesis of DL-based EEG emotion recognition from 2020–2025 is valuable. The manuscript is promising and handles an important topic, but several methodological details are insufficiently described. Addressing the required points will significantly strengthen the scientific rigor and usefulness of the review.

 Suggestions:

1. Authors can clearly articulate how this review differs from prior surveys and what unique value it adds.
2. Provide a comparative table mapping of research articles.
3. The manuscript mentions an analysis of datasets and DL approaches, but the synthesis appears descriptive rather than systematic.
4. The manuscript describes DL architectures and EEG datasets, but several essential technical details appear incomplete such as Dataset characterization, Deep learning architecture analysis and Evaluation protocols
5. The paper highlights interpretability and generalizability issues but does not thoroughly link them to the evidence provided in the reviewed papers. They can provide concrete examples of interpretability tools used in the included studies

Are the rationale for, and objectives of, the Systematic Review clearly stated?

Yes

Is the statistical analysis and its interpretation appropriate?

Not applicable

If this is a Living Systematic Review, is the ‘living’ method appropriate and is the search schedule clearly defined and justified? (‘Living Systematic Review’ or a variation of this term should be included in the title.)

Not applicable

Are sufficient details of the methods and analysis provided to allow replication by others?

Yes

Are the conclusions drawn adequately supported by the results presented in the review?

Yes

Reviewer Expertise:

Biomedical Signal and Image processing

I confirm that I have read this submission and believe that I have an appropriate level of expertise to confirm that it is of an acceptable scientific standard.

# Response

**Published:** 2026-02-17

**Authors:** Raghavendra U

**Affiliations:** Instrumentation and Control En, Manipal Institute of Technology, Manipal, Karnataka, India

**Reviewer comment:** Authors should clearly articulate how this review differs from other studies and what unique value it adds.

**Response:** We thank the reviewer for this valuable comment. We have revised the manuscript to more clearly highlight the advantages of this study and updated in Section 1.2 of the manuscript.

**Reviewer comment:**  Provide comparative table mapping of research articles.

**Response: **Thank you for this valuable comment. As the study includes a total of 233 articles, incorporating the complete table within the main manuscript was not optimal as per the journal editors. Therefore, the table has been provided as extended data and made available through an external directory. We kindly request the reviewer to refer to the Extended Data section of the manuscript for detailed information.

**Reviewer comment:** The manuscript mentions an analysis of datasets and DL approaches, but the synthesis appears descriptive rather than systematic.

**Response: **Thank you for this valuable comment.

 A Table comparing the public dataset has also been added as an external file in the directory. The Table 1 compares different datasets with respect to the stimuli used, number of EEG channels, number of subjects, and different emotion labels.

**Reviewer comment:** The manuscript lacks several essential technical details such as dataset characterization, deep learning architecture analysis and Evaluation protocols.

**Response:** Thank you for this valuable comment.

 The detailed dataset characterization has been provided in the Extended Data file, where a comprehensive comparison of the datasets is presented (refer to Table 1). Additionally, the extended data includes a structured comparison of deep learning architectures, highlighting their role in feature extraction, the types of features commonly employed, and the evaluation protocols, including subject-dependent and subject-independent settings (refer to Tables 2-4).

**Reviewer comment:** The paper highlights interpretability and generalizability issues, but does not provide concrete examples of interpretability tools used in the included studies.

**Response:** We sincerely thank the reviewer for this valuable comment. In response, the interpretability tools employed in the included studies have been highlighted in Section 4.5 of the manuscript. Additionally, we explained different interpretability methods discussing some of the studies that applied explainability and uncertainty analysis for EEG emotion recognition.

# Reviewer report: Reviewer response for version 1

**DOI:** 10.5256/f1000research.188735.r436875

**Published:** 2025-12-24

**Authors:** Brajen Kumar Deka

**Affiliations:** 1 Assam Don Bosco University, Guwahati, Assam, India

This systematic review examines 233 DL-based EEG emotion recognition studies (2020–2025) following PRISMA guidelines, focusing on datasets, stimulation methods, annotation schemes, and model architectures. It highlights emerging trends and practical needs. No statistical analysis is reported; the authors should state that a meta-analysis was not feasible due to dataset and model heterogeneity.

Are the rationale for, and objectives of, the Systematic Review clearly stated?

Yes

Is the statistical analysis and its interpretation appropriate?

No

If this is a Living Systematic Review, is the ‘living’ method appropriate and is the search schedule clearly defined and justified? (‘Living Systematic Review’ or a variation of this term should be included in the title.)

Yes

Are sufficient details of the methods and analysis provided to allow replication by others?

Yes

Are the conclusions drawn adequately supported by the results presented in the review?

Yes

Reviewer Expertise:

Speech Processing, Natural Language Processing, Image Processing, Machine Learning, Deep Learning, and Data Mining.

I confirm that I have read this submission and believe that I have an appropriate level of expertise to confirm that it is of an acceptable scientific standard.

# Response

**Published:** 2026-02-13

**Authors:** Raghavendra U

**Affiliations:** Instrumentation and Control En, Manipal Institute of Technology, Manipal, Karnataka, India

Thank you for your thoughtful review and for the insightful comments provided on our manuscript. The feedbacks have been very helpful in improving the clarity of our work. 

**Reviewer comment**: No statistical analysis was reported. The authors should state that a meta-analysis was not feasible due to dataset and model heterogeneity.

**Response to the reviewer: **We sincerely thank the reviewer for highlighting this important point. In response, we have explicitly clarified in Section 2.2 of the revised manuscript that a formal meta-analysis was not feasible due to significant heterogeneity in the datasets, model architectures, and evaluation protocols.
